# Transcriptional regulation of P63 on the apoptosis of male germ cells and three stages of spermatogenesis in mice

**DOI:** 10.1038/s41419-017-0046-z

**Published:** 2018-01-23

**Authors:** Hong Wang, Qingqing Yuan, Minghui Niu, Wenhui Zhang, Liping Wen, Hongyong Fu, Fan Zhou, Zuping He

**Affiliations:** 1 State Key Laboratory of Oncogenes and Related Genes, Renji- Med X Clinical Stem Cell Research Center, Ren Ji Hospital, School of Medicine, Shanghai Jiao Tong University, Shanghai, 200127 China; 2 Shanghai Institute of Andrology, Ren Ji Hospital, School of Medicine, Shanghai Jiao Tong University, 145 Shangdong Road, Shanghai, 200001 China; 3 Shanghai Key Laboratory of Assisted Reproduction and Reproductive Genetics, Shanghai, 200127 China; 4 Shanghai Key Laboratory of Reproductive Medicine, Shanghai, 200025 China

## Abstract

Infertility affects 10–15% of couples worldwide, and male factors account for 50%. Spermatogenesis is precisely regulated by genetic factors, and the mutations of genes result in abnormal spermatogenesis and eventual male infertility. The aim of this study was to explore the role and transcriptional regulation of P63 in the apoptosis and mouse spermatogenesis. P63 protein was decreased in male germ cells of P63^(+/−)^ mice compared with wild-type mice. There was no obvious difference in testis weight, sperm motility, and fecundity between P63^(+/−)^ and wild-type mice. However, abnormal germ cells were frequently observed in P63^(+/−)^ mice at 2 months old. Notably, apoptotic male germ cells and the percentage of abnormal sperm were significantly enhanced in P63^(+/−)^ mice compared to wild-type mice. Spermatogonia, pachytene spermatocytes and round spermatids were isolated from P63^(+/−)^ and wild-type mice using STA-PUT velocity sedimentation, and they were identified phenotypically with high purities. RNA sequencing demonstrated distinct transcription profiles in spermatogonia, pachytene spermatocytes, and round spermatids between P63^(+/−)^ mice and wild-type mice. In total, there were 645 differentially expressed genes (DEGs) in spermatogonia, 106 DEGs in pachytene spermatocytes, and 1152 in round spermatids between P63^(+/−)^ mice and wild-type mice. Real time PCR verified a number of DEGs identified by RNA sequencing. Gene ontology annotation and pathway analyzes further indicated that certain key genes, e.g., *Ccnd2*, *Tgfa*, *Hes5*, *Insl3*, *Kit*, *Lef1*, and *Jun* were involved in apoptosis, while *Dazl*, *Kit*, *Pld6*, *Cdkn2d*, *Stra8*, and *Ubr2* were associated with regulating spermatogenesis. Collectively, these results implicate that P63 mediates the apoptosis of male germ cells and regulates three stages of spermatogenesis transcriptionally. This study could provide novel targets for the diagnosis and treatment of male infertility.

## Introduction

The *P63* gene, also known as *Trp63* gene, encodes two isoforms, namely *TAp63* and *ΔNp63*, and alternative splicing at the C-terminus forms three different proteins, including α, β and γ for both *TAp63* and *ΔNp63*. It has been reported that P63 is expressed in various kinds of tissues, including heart, epidermis, urothelium, cervix, testis, kidney, thymus, and prostate in humans and mice^1^. *TAp63* mainly induces cell cycle arrest and/or apoptosis, whereas *ΔNp63* usually has an opposite effect on *TAp63*^1,2^. The α isoform of *TAp63* and *ΔNp63* is the longest one, which specifically contains the Sterile A Motif (SAM) domain that is thought to be involved in certain biological processes, e.g., development, apoptosis, and differentiation^3,4^. The β and γ isoforms of *TAp63* and *ΔNp63* have a transactivating activity, and conversely the α isoform represses transactivating activity by the C-terminal domain. It has been shown that *TAp63* gene is present in adult mouse testis whereas *ΔNp63* transcript is undetected in testis^1^. Nevertheless, other study has reported that *ΔNp63* is expressed in the testis of mice from post-natal day 1 to day 7 and from 3 to 4 weeks old^5^. At the embryo stage, P63 has been shown to balance the numbers of male germ cells by controlling germ cell apoptosis^6^. However, it remains to examine the role and molecular mechanism of P63 in regulating male germ cell development in adult mice.

Infertility affects 10–15% of couples worldwide, and male factors account for 50%. Spermatogenesis is a complex process that includes three main stages, namely the mitosis of spermatogonia, meiosis of spermatocytes, and spermiogenesis of spermatids. Spermatogenesis is precisely regulated by genetic factors, and the mutations of genes result in abnormal spermatogenesis and eventual male infertility. Since P63 homogeneous mutant mice die within several hours after birth due to maternal neglect and dehydration^7,8^, P63^(+/−)^ adult mice were thus utilized in this study to probe the function and transcriptional regulation of P63 in three stages of mammalian spermatogenesis. P63 mutation was generated by the pTV12E(60) vector that was integrated into *p63* locus to produce a recombinant allele, namely *p63*^Brdm2^^7^. STA-PUT velocity sedimentation was used to effectively separate spermatogonia, pachytene spermatocytes and round spermatids from wild-type mice and P63^(+/−)^ mice. We and peers have demonstrated that it is feasible to obtain various kinds of male germ cells with high purity and viability in humans and mice via STA-PUT velocity sedimentation^9–11^. STA-PUT approach can be effectively used to separate cells in terms of the unit gravity based upon cell size and mass via a linear BSA gradient and sedimentation velocity^12–14^. Compared with other techniques, e.g., fluorescence-activated cell sorting (FACS), magnetic-activated cell sorting (MACS) and elutriation, STA-PUT approach has several advantages in obtaining each specific cell type of male germ cells, including high purity and viability of these cells^11^. In this study, we have for the first time demonstrated that P63 mutation leads to the increases in apoptosis of male germ cells and abnormal sperm. Furthermore, we have unveiled a large scale of distinct transcription profiles in spermatogonia, pachytene spermatocytes, and round spermatids between P63^(+/^^−^^)^ mice and wild-type mice. This study thus sheds a novel insight into the function and mechanisms of P63 in mediating the apoptosis of male germ cells and mammalian spermatogenesis and it offers novel targets for treating male infertility.

## Results

### Genotype of the P63^(+/^^−^^)^ mice

**Fig. 1:**
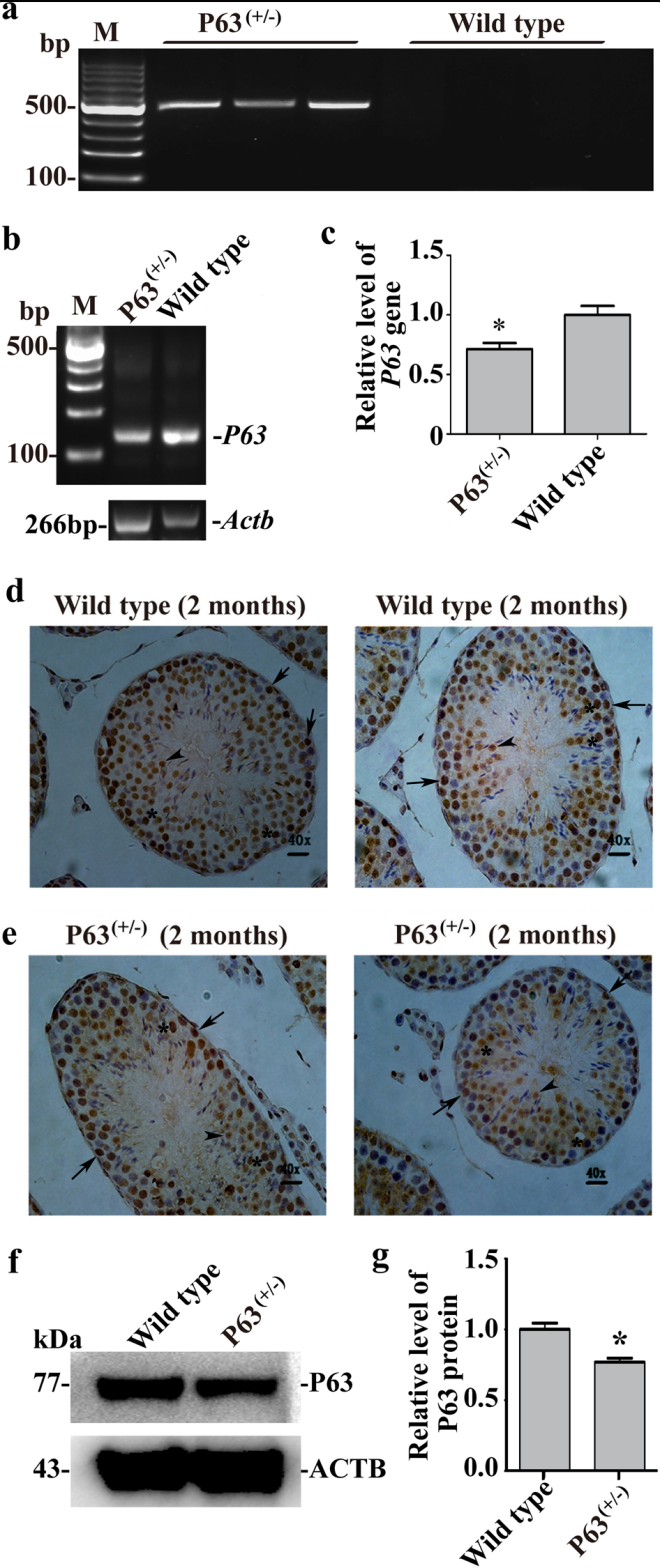
Genotype and the expression of P63 protein in P63^(+/^^−^^)^ mice and wild-type mice PCR showed the DNA fragment of *p63*^Brdm2^ in P63^(+/^^−^^)^ mice and wild-type mice **a**. PCR indicated the expression of *P63* gene in P63^(+/^^−^^)^ male mice and wild-type mice **b**–**c**. Immunohistochemistry revealed the protein expression of P63 in the testis sections from wild-type mice **d** and P63^(+/^^−^^)^ mice **e**. Scale bars in **d**–**e** = 20 μm. Western bolts demonstrated the protein expression of P63 in male germ cells of P63^(+/^^−^^)^ mice and wild-type mice **f**. ACTB served as a loading control of total proteins. The relative expression level of P63 in male germ cells of P63^(+/^^−^^)^ mice and wild-type mice after normalization to ACTB **g**. * indicated statistically significant differences (*p* < 0.05) between the P63^(+/^^−^^)^ mice and wild-type mice

The genotype of the offspring of the P63^(+/^^−^^)^ male mice and wild-type (C57BL/6) female mice was identified using PCR. We observed that the amplified genomic DNA fragments of *p63*^Brdm2^ were present in P63^(+/^^−^^)^ male mice but undetected in wild-type female mice (Fig. 1a). PCR showed that the level of *P63* gene was lower in P63^(+/^^−^^)^ male mice than wild-type mice (Fig. 1b, c). We chose an antibody that specially recognized all P63 isoforms to locate P63 protein in mouse testes. Immunohistochemistry revealed that P63 protein was expressed in the nuclei of spermatogonia (arrows), spermatocytes (asterisks), and round spermatids (arrowheads) in wild-type male mice (Fig. 1d) and the P63^(+/^^−^^)^ male mice (Fig. 1e). Furthermore, western bolts demonstrated that the expression level of the P63 protein was decreased by 23.1% ± 3.4% in male germ cells of the P63^(+/^^−^^)^ mice compared to wild-type mice (Fig. 1f, g). These results suggest that P63 mutation leads to the reduction of P63 protein in male mice.

### Phenotypic characteristics of the P63^(+/^^−^^)^ mice and wild-type mice

**Fig. 2:**
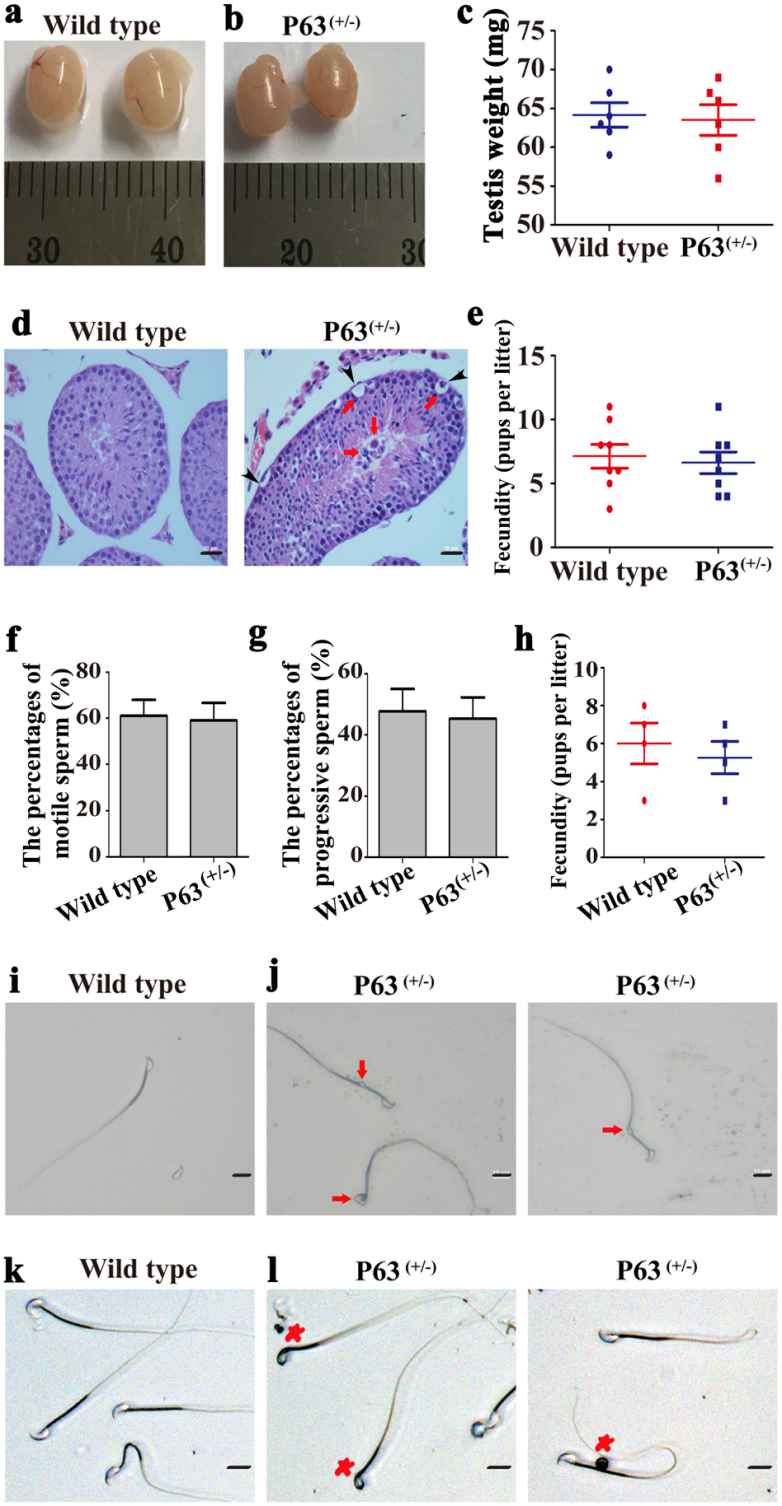
The phenotypic characterization of the P63^(+/^^−^^)^ mice and wild-type mice The images of testes from wild-type mice **a** and P63^(+/^^−^^)^ mice **b** at 2 months old. The testis weight of wild-type mice and P63^(+/^^−^^)^ mice at 2 months old (*n* = 6) **c**. H&E staining of testis sections from wild-type mice (**d**, left panel) and P63^(+/^^−^^)^ mice (**d**, right panel) at 2 months old. Scale bar in **d** = 20 μm. The numbers of pups per litter of wild-type mice and P63^(+/^^−^^)^ male mice at 2–8 months old crossed with wild-type female mice **e**. The percentages of motile sperm **f** and progressive sperm **g** in wild-type mice and P63^(+/^^−^^)^ mice at 2 months old. All data of motility measurements were presented from at least three independent experiments. The fecundity of P63^(+/^^−^^)^ mice and wild-type mice at round 12 months **f**. The morphology of sperm of wild-type mice **i** and P63^(+/^^−^^)^ mice **j** at 2 months old under a phase-contrast microscope. H&E staining of sperm from wild-type mice **k** and P63^(+/^^−^^)^ mice **l** at 2 months old. Scale bars in **i**–**l** = 20 μm

**Fig. 3:**
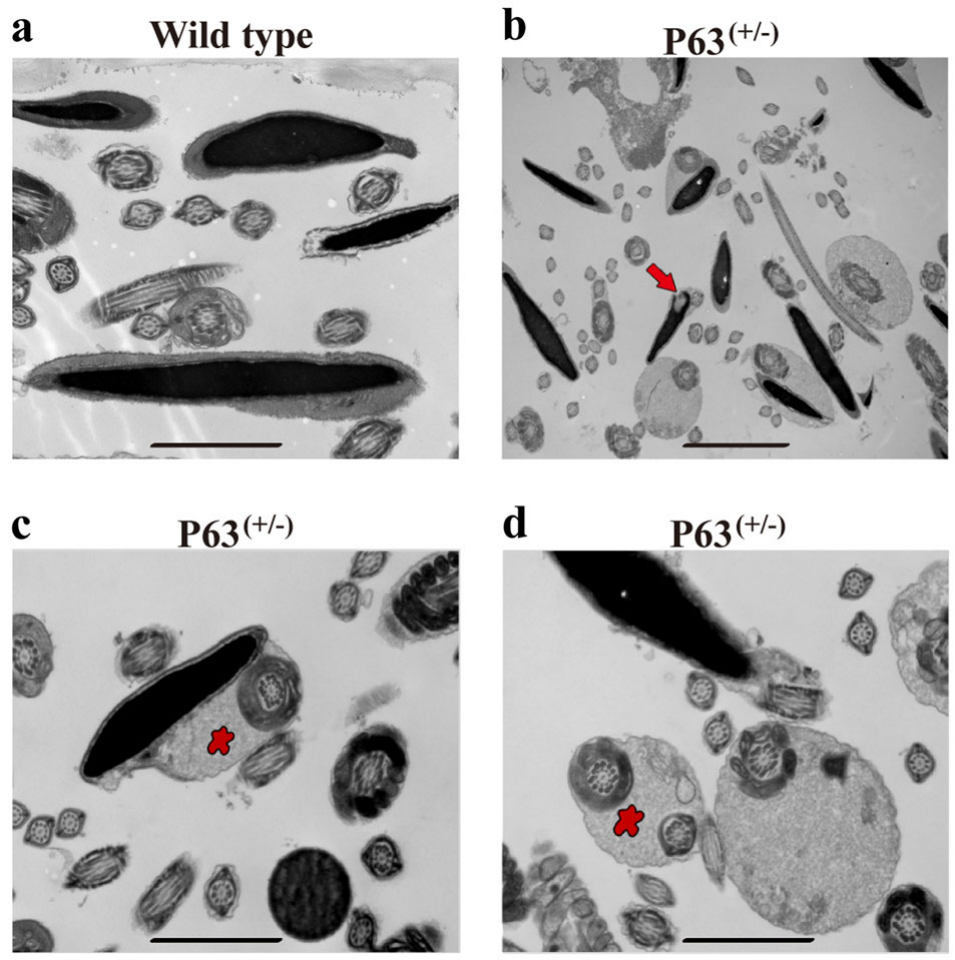
Ultrastructure of sperm in cauda epididymis from P63^(+/^^−^^)^ mice and wild-type mice at 2 months old TEM showed the ultrastructure of sperm in wild-type mice **a** and P63^(+/^^−^^)^ mice **b**–**d**. **a**: normal ultrastructure; **b** vacuoles in nuclei (arrow); **c** abnormal cytoplasm (asterisk); **d** multiple tails (asterisk). Scale bars in **a**, **c**–**d** = 0.5 μm, and scale bar in **b** = 1 μm

We next evaluated the phenotypic characteristics of the P63^(+/^^−^^)^ mice and wild-type mice. There was no obvious difference in the size and weight of testes between the P63^(+/^^−^^)^ mice (63.5 ± 4.9 mg, *P* = 0.80, *n* = 6) and the wild-type mice (64.2 ± 3.9 mg, *P* = 0.80, *n* = 6) (Fig. 2a–c). However, H&E staining showed that abnormal and larger male germ cells (arrows) frequently existed in seminiferous tubules especially in the lumen of the P63^(+/^^−^^)^ mice (Fig. 2d, right panel), while normal male germ cells were found in the testes of wild-type mice (Fig. 2d, left panel). Meanwhile, we found atrophy (arrowheads) of seminiferous tubule epithelium in the P63^(+/^^−^^)^ mice (Fig. 2d, right panel). There was no significant difference in the fecundity (6.6 ± 2.4 vs. 7.1 ± 2.6, *P* = 0.70, *n* = 8) (Fig. 2e), sperm motility (59.0% ± 13.2% vs. 61.0% ± 12.1%, *P* = 0.86, *n* = 3) (Fig. 2f), or the percentages of progressive motility sperms (45.0% ± 15.0% vs. 47.7% ± 12.7%, *P* = 0.83, *n* = 3) (Fig. 2g) between the P63^(+/^^−^^)^ mice and wild-type mice. The motility of sperm can be classified as non-motile, non-progressive motile, and progressive motile. Non-motile and non-progressive motile spermatozoa lead to male infertility, whereas progressive motile spermatozoa have the potential of fecundity^15^. Furthermore, the fecundity was reduced from 6.0 ± 2.2 (*n* = 4) in wild-type mice to 5.3 ± 1.7 (*n* = 4) in P63^(+/^^−^^)^ mice at round 12 months (Fig. 2h) in spite of no statistical difference between these two groups, since P63^(+/^^−^^)^ mice are heterozygous mutation rather than homozygous mutation. Notably, phase-contrast microscope and hematoxylin-eosin **(**H&E) staining demonstrated that there were a number of abnormal sperm, e.g., round-headed spermatozoa (asterisks), cytoplasmic droplet (arrows), and aberrant sperm tails (20.8% ± 4.3%) in the P63^(+/^^−^^)^ mice (Fig. 2j, l), while sperm with normal morphology were found in wild-type mice (Fig. 2i, k). Moreover, transmission electron microscopy (TEM) displayed that nucleus vacuoles (Fig. 3b, arrow), abnormal cytoplasm (Fig. 3c, asterisk), and multiple tails (Fig. 3d, asterisk) were seen in sperm of the P63^(+/^^−^^)^ mice (27.3% ± 3.6%) compared to wild-type mice (9.8% ± 3.3%) (Fig. 3a). Collectively, these data indicate that P63 mutation results in abnormalities in morphology and ultrastructure of sperm in mice.

### P63 mutation results in more apoptotic cells in mice

**Fig. 4:**
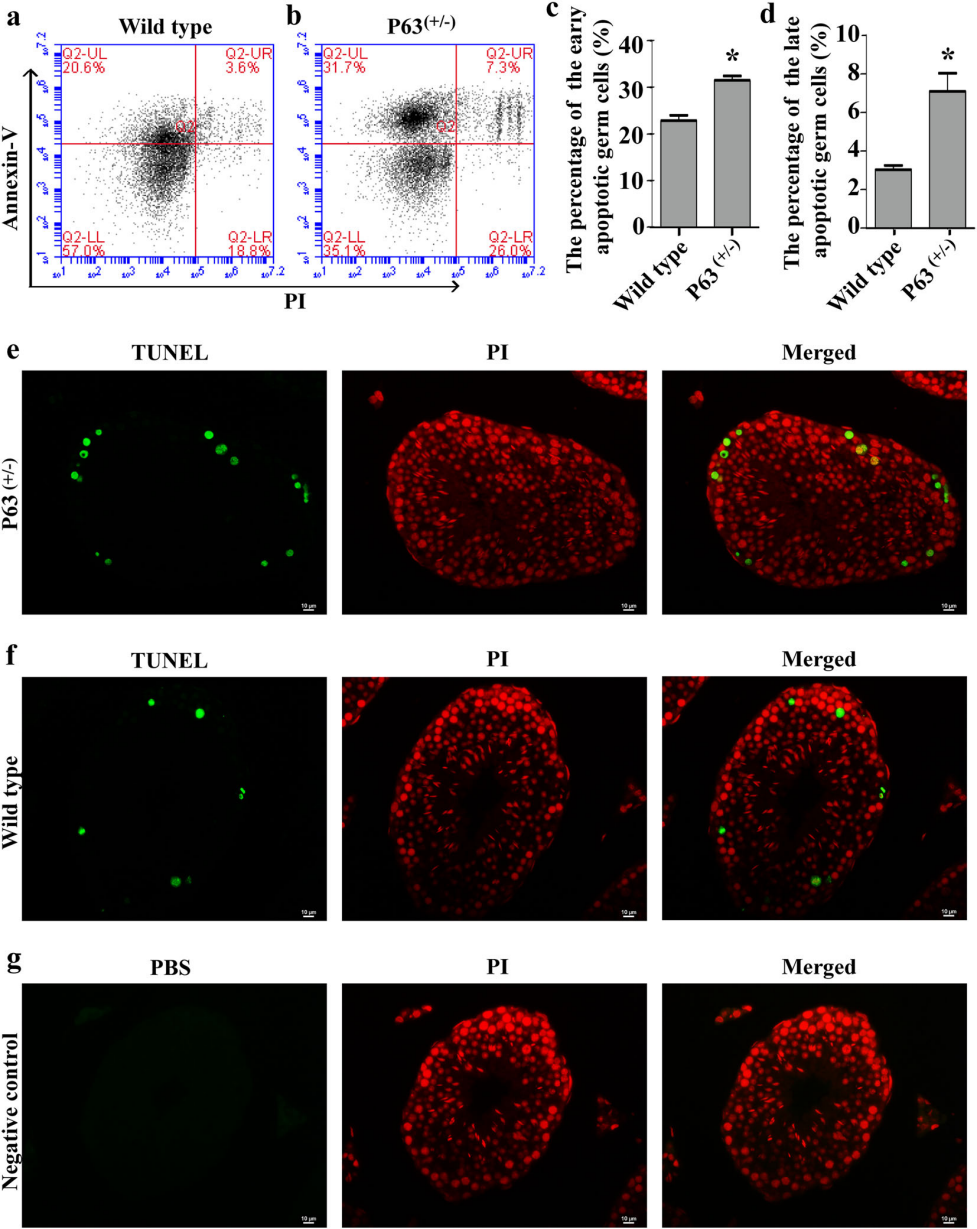
The apoptosis in male germ cells of the P63^(+/^^−^^)^ mice and wild-type mice The percentages of apoptotic male germ cells in wild-type mice **a** and P63^(+/^^−^^)^ mice **b** at 2 months old. The percentages of early apoptosis **c** and late apoptosis **d** in male germ cells of wild-type mice and P63^(+/^^−^^)^ mice were calculated using Student’s *t*-test. * indicated statistically significant differences (*p* < 0.05). TUNEL assay demonstrated the TUNEL-positive cells (green fluorescence) in P63^(+/^^−^^)^ mice **e** and wild-type mice **f**. Replacement the TdT enzyme with PBS was used as the negative control **g**. PI (red fluorescence) was used to label cell nuclei. Scale bars in **e**–**g** = 10 μm. The data in Fig. 4a–g were presented from three independent experiments

Annexin V-FITC/propidium iodide (PI) staining and flow cytometry was used to compare the apoptosis of male germ cells in the P63^(+/^^−^^)^ mice and the wild-type mice by two-step enzymatic digestion and the differential plating. As shown in Fig. 4a–c, the percentage of the early apoptotic male germ cells was enhanced from 22.9% ± 2.0% to 31.5% ± 1.7% in P63^(+/^^−^^)^ mice compared with wild-type mice. In addition, the percentage of the late apoptotic male germ cells was increased from 3.0% ± 0.4% to 7.1% ± 1.6% in P63^(+/^^−^^)^ mice compared to wild-type mice (Fig. 4a, b, d). The results suggest that P63 mutation leads to more apoptotic cells in mice. TUNEL assay further revealed that the percentage of TUNEL-positive cells were increased from 2.3% ± 0.5% to 4.1% ± 0.5% in P63^(+/^^−^^)^ mice (Fig. 4e) compared to wild-type mice (Fig. 4f). These results suggest that P63 mutation leads to more apoptosis in mouse testis in vivo.

### Isolation and identification of mouse spermatogonia, pachytene spermatocytes, and round spermatids from the P63^(+/^^−^^)^ mice and wild-type mice

**Fig. 5:**
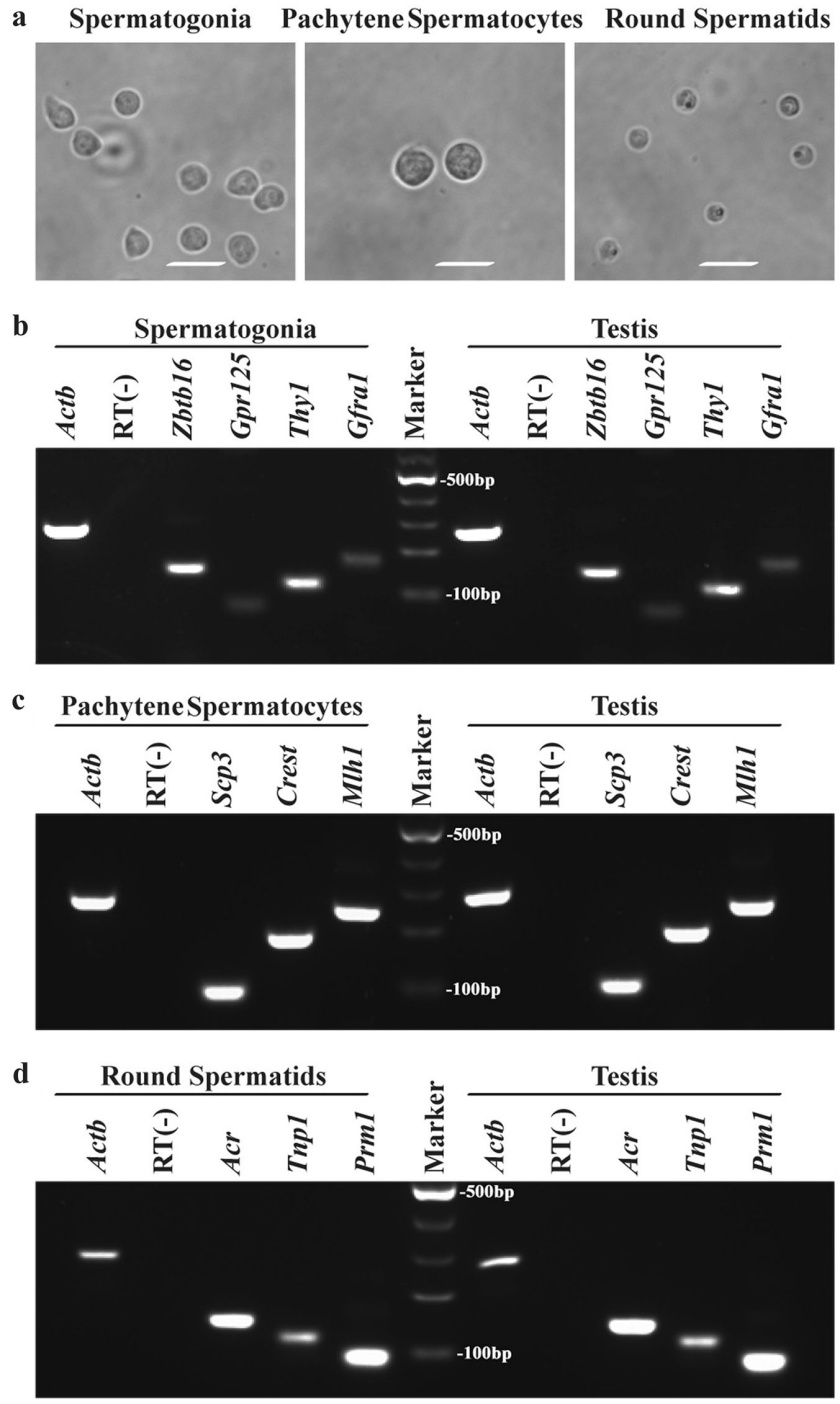
The morphological and phenotypic characteristics of mouse spermatogonia, pachytene spermatocytes and round spermatids Phase-contrast microscope showed the morphological characteristics of the freshly isolated spermatogonia, pachytene spermatocytes and round spermatids **a**. Scale bars in **a** = 20 μm. RT-PCR revealed the transcripts of *Gfra1, Thy1, Gpr125*, and *Zbtb16* in the freshly isolated mouse spermatogonia and mouse testis **b**, *Mlh1, Crest*, and *Scp3* in the freshly isolated pachytene spermatocytes and mouse testis **c**, *Acr, Prm1*, and *Tnp1* in the freshly isolated round spermatids and mouse testis **d**. *Actb* was employed as a loading control of total RNA, and RNA samples without RT (RT−) but with PCR by *Actb* primers were used as negative controls

STA-PUT apparatus via the velocity sedimentation was used to isolate spermatogonia, pachytene spermatocytes, and round spermatids from P63^(+/^^−^^)^ mice and wild-type mice. The isolated cells were identified according to their morphological characteristics and phenotype using specific gene and protein markers for these cells. Mouse spermatogonia were collected by cell fractions 35–50, and under the phase-contrast microscope, they were spherical in morphology with large and round nuclei and a diameter ranging from 10 to 12 μm (Fig. 5a, left panel). Pachytene spermatocytes were obtained by cell fractions 5–25, and these cells had patchy condensed chromatin with the diameter ranging from 14 to 18 μm (Fig. 5a, middle panel). Meanwhile, round spermatids were collected from 60 to 75, and they were smaller cells with round nuclei with a diameter ranging from 8 to 10 μm (Fig. 5a, right panel). A number of gene markers for spermatogonia, pachytene spermatocytes, and round spermatids were chosen to assess the phenotype of the freshly isolated cells using RT-PCR. We found that the transcripts of *Gpr125*, *Gfra1*, *Thy1*, and *Zbtb16* (also known as *Plzf*) were expressed in isolated spermatogonia (Fig. 5b), and *Scp3, Crest*, and *Mlh1* mRNA was detected in isolated pachytene spermatocytes (Fig. 5c). Additionally, the transcription of *Acr, Tnp1*, and *Prm1* was present in isolated round spermatids (Fig. 5d). The expression of the genes mentioned above in mouse testis served as positive controls, and RNA without RT (RT-) but with PCR was utilized as a negative control. In contrast, the transcripts of *Scp3, Crest, Mlh1, Acr, Tnp1*, and *Prm1* were not detected in isolated spermatogonia (Supplemental Fig. 1a), and *Gpr125, Gfra1, Zbtb16, Acr, Tnp1*, and *Prm1* were undetected in isolated pachytene spermatocytes (Supplemental Fig. 1b). The transcription of *Gpr125, Gfra1, Zbtb16, Scp3, Crest*, and *Mlh1* was not detected in isolated round spermatids (Supplemental Fig. 1c).

**Fig. 6:**
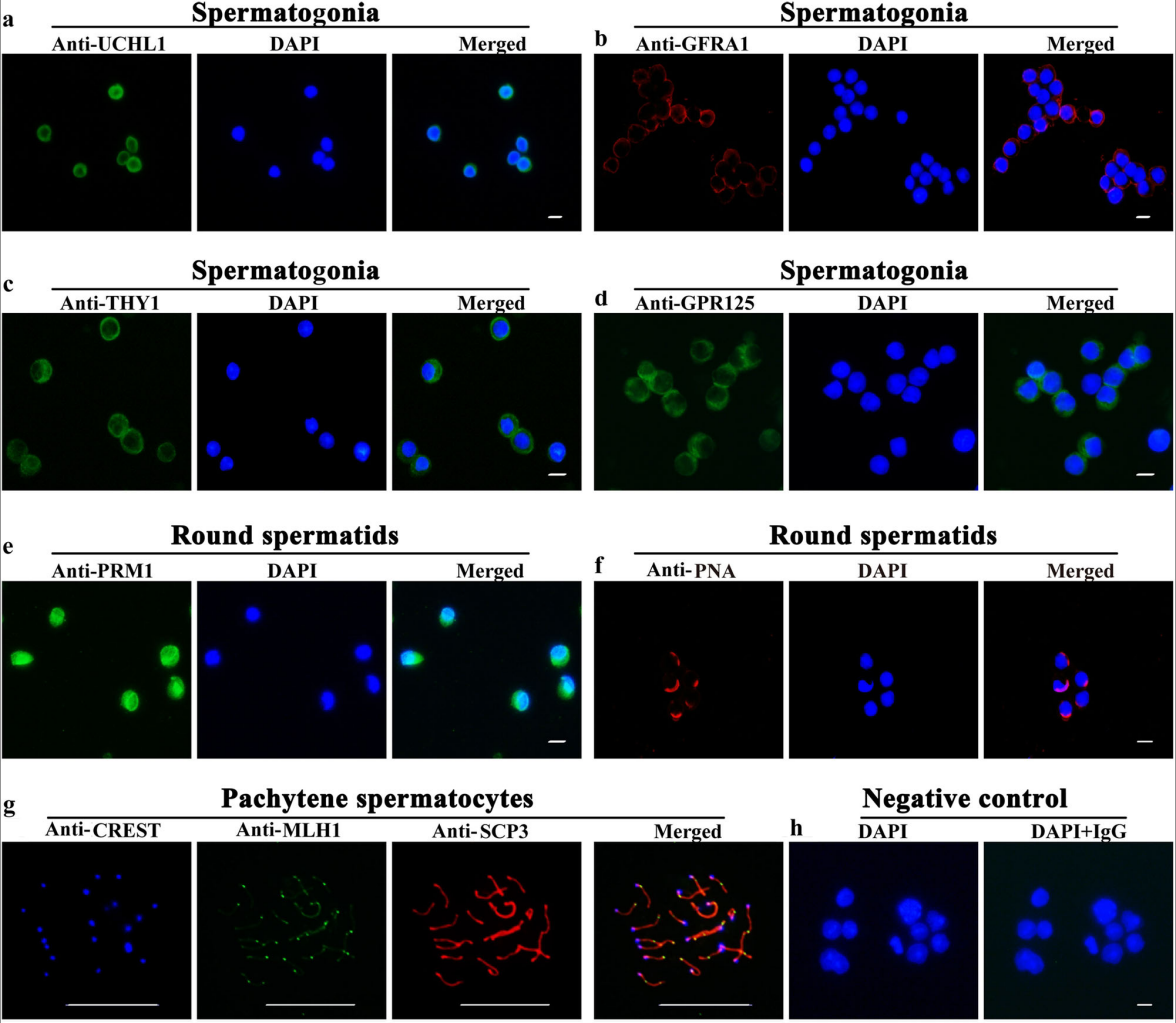
The identification of the freshly isolated spermatogonia, pachytene spermatocytes, and round spermatids Immunocytochemistry showed the protein expression of UCHL1 **a**, GFRA1 **b**, THY1 **c**, and GPR125 **d** in the freshly isolated mouse spermatogonia. Immunocytochemistry demonstrated the protein expression of PRM1 **e** and PNA **f** in the isolated mouse round spermatids. Meiosis spread assays by triple immunostaining revealed the expression of CREST (blue fluorescence), MLH1 (green fluorescence) and SCP3 (red fluorescence) in the freshly isolated mouse pachytene spermatocytes **g**. The data shown in **a**–**g** were representatives from three independent experiments. Replacement of primary antibodies with isotype IgGs in male germ cells served as a negative control **h**. Scale bars in **a**–**h** = 10 μm

Furthermore, immunocytochemistry was performed to identify the purity of spermatogonia and round spermatids. As shown in Fig. 6, more than 90% of isolated cells were positive for UCHL1 (Fig. 6a), GFRA1 (Fig. 6b), THY1 (Fig. 6c), and GPR125 (Fig. 6d), markers for spermatogonia and SSCs^16^. In total, over 95% of haploid cells were stained positively for PRM1 (Fig. 6e) and PNA (Fig. 6f), hallmarks for round spermatids^11^. Meiosis spread was conducted to identify the isolated pachytene spermatocytes. As shown in Fig. 6g, more than 90% of tetraploid cells were co-expressing CREST (blue fluorescence), MLH1 (green fluorescence), and SCP3 (red fluorescence), markers for spermatocytes^11^. Replacement of primary antibodies with isotype IgGs served as negative controls, and no immunostaining was observed in male germ cells (Fig. 6h), thus verifying the specific immunostaining of these antibodies mentioned above. Immunocytochemistry demonstrated that the immunostaining of SCP3 and PRM1 was not seen in isolated spermatogonia (Supplemental Fig. 2a), and UCHL1 and PRM1 were not detected in isolated pachytene spermatocytes (Supplemental Fig. 2b). The expression of UCHL1 and SCP3 was not observed in isolated round spermatids (Supplemental Fig. 2c). These data further verified the high purity of the isolated spermatogonia, pachytene spermatocytes, and round spermatids. In addition, the viability of these freshly isolated cells was over 95%, as assessed with trypan blue exclusion assay (data not shown).

### Distinct global transcription profiles in spermatogonia, pachytene spermatocytes, and round spermatids between the P63^(+/^^−^^)^ mice and wild-type mice

**Fig. 7:**
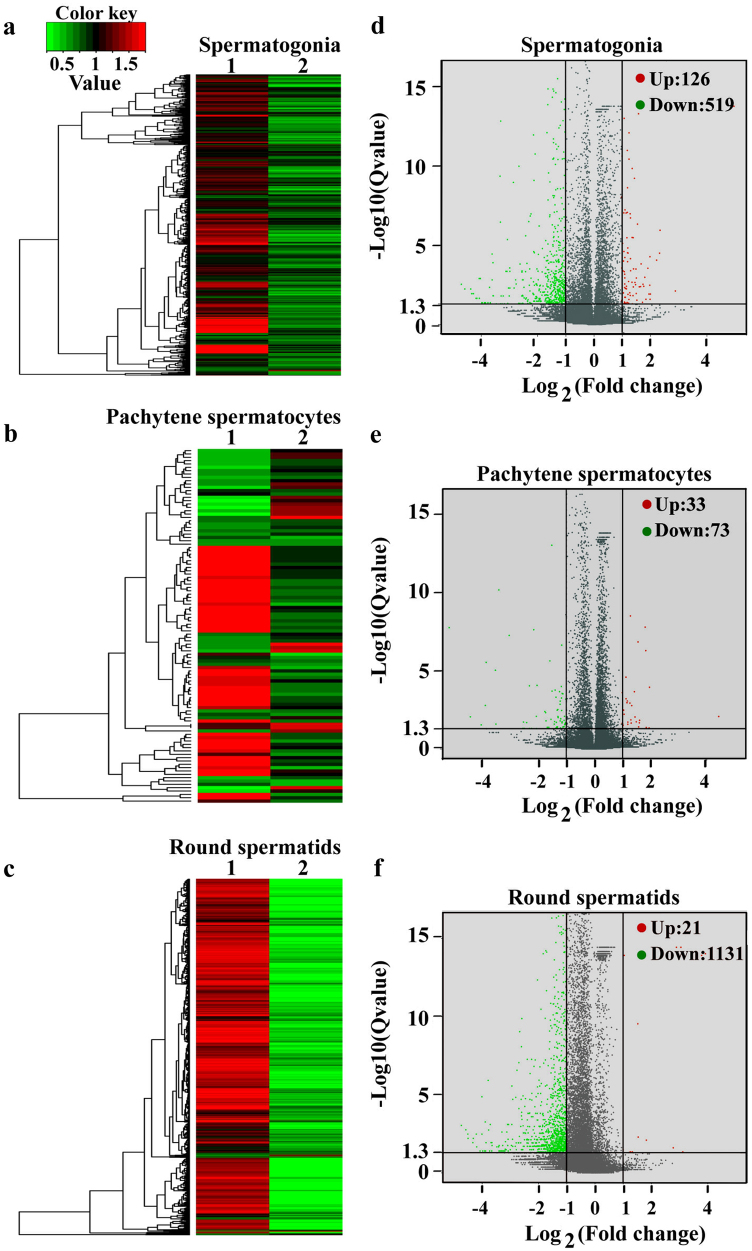
RNA sequencing revealed distinct global transcription profiles in spermatogonia, pachytene spermatocytes and round spermatids between P63^(+/^^−^^)^ mice and wild-type mice Hierarchical clustering analysis revealed the differentially expressed genes (DEGs) in spermatogonia **a**, pachytene spermatocytes **b** and round spermatids **c** between P63^(+/^^−^^)^ mice and wild-type mice. Notes: ‘1’ indicated wild-type mice and ‘2’ denoted P63^(+/^^−^^)^ mice. Volcano plots showed the DEGs in spermatogonia **d**, pachytene spermatocytes **e** and round spermatids **f** between P63^(+/^^−^^)^ mice and wild-type mice. The log_2_ scales of the expression signal values were plotted for all probes. Standard selection criteria to identify P63^(+/^^−^^)^ mice and wild-type mice was established at log_2_ (fold change)>1 and *p*-value <0.05. Upregulated and downregulated genes were represented by red and green color dots, respectively

To gain a novel insight into the molecular mechanisms of P63 in regulating the apoptosis of male germ cells and three stages of spermatogenesis, RNA sequencing was performed to compare the large scale of transcription profiles in spermatogonia, pachytene spermatocytes,and round spermatids between the P63^(+/^^−^^)^ mice and wild-type mice. Total RNA was extracted from these freshly isolated cells, and its quality was evaluated for RNA sequencing using electropherogram (Supplemental Fig. 3a–c) to ensure high quality. The total reads and gene numbers in spermatogonia, pachytene spermatocytes, and round spermatids of the P63^(+/^^−^^)^ mice and wild-type mice were shown in Table S2. Compared with wild-type mice, 519 genes were downregulated and 126 genes upregulated in spermatogonia of the P63^(+/^^−^^)^ mice (Fig. 7a, d). In total, 73 genes were downregulated while 33 genes were upregulated in pachytene spermatocytes of the P63^(+/^^−^^)^ mice compared to wild-type mice (Fig. 7b, e). In addition, 1131 genes were downregulated and 21 genes were upregulated in round spermatids of P63^(+/^^−^^)^ mice compared with wild-type mice (Fig. 7c, f). Together, these data implicate that P63 regulates three main stages of spermatogenesis transcriptionally.

### Verification of RNA sequencing data by quantitative real-time PCR

**Fig. 8:**
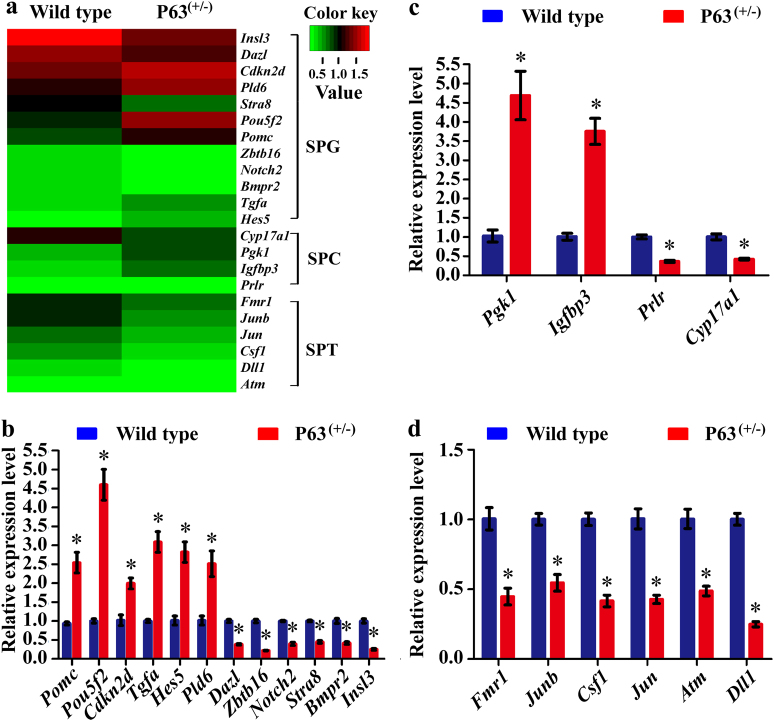
The DEGs in spermatogonia, pachytene spermatocytes and round spermatids between P63^(+/^^−^^)^ mice and wild-type mice Hierarchical clustering analysis showed 12 DEGs in spermatogonia (SPG), 4 DEGs in pachytene spermatocytes (SPC), and 6 DEGs in round spermatids (SPT) between P63^(+/^^−^^)^ mice and wild-type mice **a**. Real-time PCR verified 12 DEGs in spermatogonia **b**, 4 DEGs in pachytene spermatocytes **c** and 6 DEGs in round spermatids **d** between P63^(+/^^−^^)^ mice and wild-type mice. * indicated statistically significant differences (*p* < 0.05) between P63^(+/^^−^^)^ mice and wild-type mice. The data in Fig. 8b–d were presented from three independent experiments

To verify the results of RNA sequencing, quantitative real time PCR (Q-PCR) was conducted with three independent experiments. In total, 22 differentially expressed genes (DEGs) that were  involved in apoptosis and spermatogenesis in mouse spermatogonia, pachytene spermatocytes and round spermatids were chosen randomly for verification. RNA sequencing showed the different expression levels of these DEGs in spermatogonia, pachytene spermatocytes, and round spermatids between P63^(+/^^−^^)^ mice and wild-type mice (Fig. 8a). Real-time PCR further demonstrated the transcripts of 12 DEGs in spermatogonia (Fig. 8b), 4 DEGs in pachytene spermatocytes (Fig. 8c) and 6 DEGs in round spermatids (Fig. 8d) between P63^(+/^^−^^)^ mice and wild-type mice. Specifically, the transcription of *Pomc, Pou5f2, Cdkn2d, Tgfa, Hes5, Pld6, Pgk1*, and *Igfbp3* was statistically upregulated whereas the transcripts of *Dazl, Zbtb16, Notch2, Stra8, Bmpr2, Insl3, Prlr, Cyp17a1, Fmrl, Junb, Csf1, Jun, Atm*, and *Dll1* were statistically downregulated in spermatogonia, pachytene spermatocytes, and round spermatids of P63^(+/^^−^^)^ mice compared with wild-type mice, which were completely consistent with the RNA sequencing results.

**Fig. 9:**
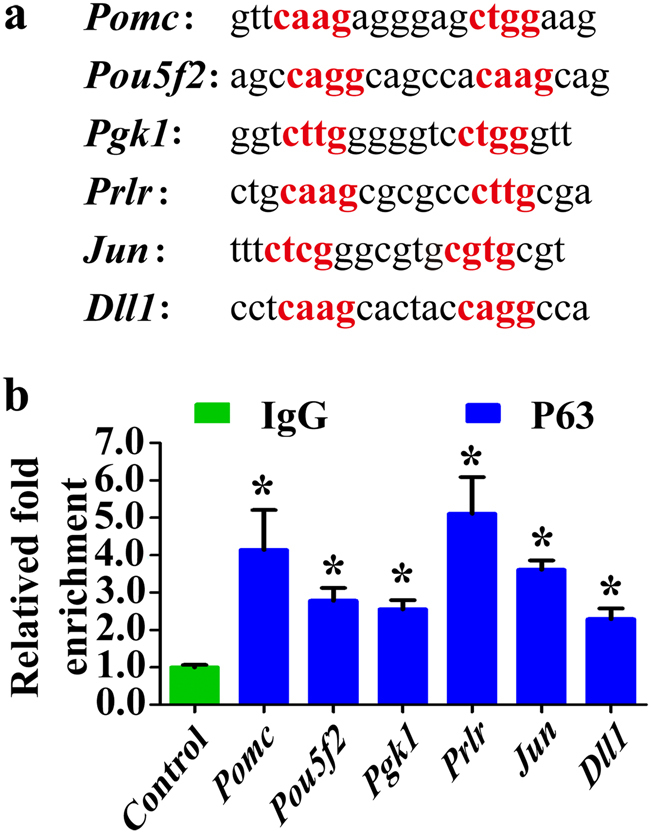
ChIP-qPCR analysis of P63 and *Pomc*, *Pou5f2*, *Pgk1*, *Prlr*, *Jun*, and *Dll1* genes in male mice P63-binding sites on *Pomc*, *Pou5f2*, *Pgk1*, *Prlr*, *Jun*, and *Dll1* promoter regions **a**. The nucleotides in red fonts correspond to the core nucleotide sequence required for P63-binding **a**. ChIP was performed using P63 antibody, and IgG was used as the control. Real-time PCR was executed on the promoter sequences **b**. * indicated statistically significant differences (*p* < 0.05)

To unveil the relationship between P63 and the DEGs, ChIP-qPCR assay was conducted and demonstrated that P63 protein directly regulated *Pomc*, *Pou5f2*, *Pgk1*, *Prlr*, *Jun*, and *Dll1* genes (Fig. 9b).

### Gene ontology and pathway analysis of the DEGs in spermatogonia, pachytene spermatocytes, and round spermatids between the P63^(+/^^−^^)^ mice and wild-type mice

DAVID software (https://david.ncifcrf.gov) was used to perform gene ontology (GO) annotation on DEGs of spermatogonia, pachytene spermatocytes and round spermatids from P63^(+/^^−^^)^ mice and wild-type mice. As shown in Table S3, there were 22 genes that were involved in the regulation of spermatogenesis, 28 genes that negatively controlled apoptosis, and 21 mRNAs that were related to the positive regulation of apoptosis in spermatogonia. There were 6 genes involved in the steroid biosynthetic process, 8 genes that were associated with the regulation of oxidation-reduction process, and 4 genes in the positive regulation of apoptosis in pachytene spermatocytes (Table S4). In total, 31 genes were involved in the regulation of apoptotic process, and 24 genes were associated with the positive regulation of apoptotic process in round spermatids; 10 genes were involved in the cell cycle arrest process, and 6 genes were related to sperm tail morphogenesis in round spermatids (Table S5).

Kyoto encyclopedia of genes and genomes (KEGG) pathway analysis was further carried out to elaborate the molecular mechanisms underlying the apoptosis and spermatogenesis. The detailed information of numerous signaling pathways was shown on the DEGs of spermatogonia (Table S6), pachytene spermatocytes (Table S7), and round spermatids (Table S8) between P63^(+/^^−^^)^ mice and wild-type mice, which sheds novel insights into molecular mechanisms of P63 in mediating three stages of spermatogenesis.

## Discussion

P53 family is composed of three transcription factors, namely P53, P63, and P73^17–19^. In structure, P53, P63, and P73 are highly homologous in sequence. It has been showed that all three members of P53 family are expressed in mouse testis^20^. P53 plays an important role in the maintenance of genomic integrity in mammal, and it is involved in a variety of cellular functions, including the regulation of cell cycle, apoptosis, and DNA repair. It has been reported that P73 mutation reduces the proliferation and increases the apoptosis of male germ cells^21^. P63 is expressed in numerous tissues, including heart, epidermis, urothelium, cervix, testis, kidney, adrenal, thymus, and prostate, in humans and mice^1^. Here we have for the first time demonstrated that P63 mutation leads to the enhancement of early and late apoptosis of male germ cells in adult mice.

Spermatogenesis consists of the division of spermatogonia, meiosis of spermatocytes, and spermiogenesis of spermatids, which is regulated by various kinds of genetic and epigenetic factors. P63 protein is expressed during primordial germ cell (PGC) migration through hindgut epithelium and dorsal mesentery at E8.5–E11.5^22^. P63 is continuously translated in female germ cells during meiotic arrest, and it protects the oocytes from apoptosis induced by irradiation^23,24^. In male, P63 protein is localized in the nuclei of mouse testicular germ cells of embryos at different stages^5,25^. We found that P63 protein was present in the nuclei of spermatogonia, spermatocytes and round spermatids in adult wild-type mice and adult P63^(+/^^−^^)^ mice and that P63 mutation resulted in the decrease of P63 protein in adult mice. Although there was no remarkable difference in the size and weight of testes, fecundity, sperm motility, or progressive motility sperms between the P63^(+/^^−^^)^ mice and the wild-type mice, abnormally larger male germ cells were observed in seminiferous tubules of the P63^(+/^^−^^)^ mice, suggesting that P63 mutation causes the abnormality of male germ cells. These abnormal cells might be due to either incomplete differentiation or maturation of germ cells or a product of apoptosis, as evidenced by our morphologic and ultrastructure observation, which were similar to those cells in Bax or P53-null mouse testis^26–28^.

Significantly, we found that the percentages of both early and late apoptotic germ cells were increased in P63^(+/^^−^^)^ mice compared to wild-type mice, implicating that P63 mutation leads to cellular apoptosis in adult male mice. We identified, using RNA sequencing and real-time PCR, numerous DEGs that were involved in apoptosis, e.g., *Insl3, Apc, Jun, Atm, Rara, Kitl, Lef1, Tgfa, Notch2*, and *Ccnd2*. As examples, *Insl3*, a member of the insulin superfamily, is secreted by Leydig cells of both fetal and adult mammalian testis^29,30^. In Insl3^−^^/^^−^ mice, the testicular development process and the epididymis are abnormal^31,32^. There are normal development in the testis and epididymis of P63^(+/^^−^^)^ mice, which might be due to the fact that these mice are heterozygous mutation but not homozygous mutation. In boar testes, the apoptotic germ cells are increased by about fourfold after neutralizing INSL3 via up-regulating the pro-apoptotic CASP3 and BAX and downregulating the anti-apoptotic XIAP and BCL2^33^. *Apc* is recognized as a substrate of *Casp3* and it plays a key role in maintaining cellular homeostasis by the apoptotic surveillance^34–36^. In the AhCre Apc^(fl/fl)^ adult mice, the frequency of apoptotic male mouse germ cells is increased, whereas pachytene spermatocytes, round spermatids, and elongating spermatids are statistically decreased^37^. In spermatogenesis, ataxia telangiectasia-mutated (Atm) plays a pivotal role in both premeiotic spermatogonial cells and premeiotic germ cell maintenance^38,39^, and it regulates cell cycle and apoptosis in response to DNA double-strand breaks (DSB), oxidative stress, and telomere eosin^40,41^. The Atm homozygous mutation mice are infertile, and the seminiferous tubules are completely devoid of mature gametes. Meanwhile, spermatogonia assume cell cycle arrest and are eliminated by apoptosis in Atm-null mice. In this study, the transcription of *Atm* was reduced in P63^(+/^^−^^)^ mice, which was consistent with the fact that the early and late apoptotic germ cells were increased by P63 heterozygous mutation.

We also unveiled, using RNA sequencing and real-time PCR, a number of DEGs (e.g., *Dazl, Zbtb16, Stra8*, and *Pou5f2*) that were associated with regulating the spermatogenesis. *Zbtb16*, also known as *Plzf*, is a hallmark for human and mouse spermatogonial stem cells, and it plays a crucial role in maintaining spermatogonial self-renewal. Silencing the *Zbtb16* function leads to spermatogonial differentiation^42,43^. The self-renewal capability of spermatogonial stem cells is reduced progressively in mice lacking *Zbtb16*, and an increase in apoptosis and loss of seminiferous tubule structure occur^42^. Deleted in azoospermia-like (DAZL) protein, a homolog of the deleted in azoospermia (DAZ), is expressed in the nuclei and cytoplasm of germ cells at all stages of spermatogenesis in humans, mice, and pigs^44–47^. The abnormal expression of DAZL affects about 10% of males with azoospermia or oligozoospermia^48–50^. Although the heterozygous (Dazlα^Tm1Hgu/+^) male mice are fertile, about 60% of the sperm is visibly abnormal, and there is no sperm in the Dazlα^Tm1Hgu/Tm1Hgu^ epididymid. Stimulated by retinoic acid 8 (*Stra8*) induced by retinoic acid (RA) can trigger the onset of meiosis both in male and female gonads^51,52^. In males, *Stra8* is firstly expressed in spermatogonia at post-natal day 5, and its level is increased in premeiotic spermatocytes^53^. It has been reported that *Stra8* is dispensable for the meiotic initiation and required for the progression of the meiotic prophase. Spermatogenesis is severely disrupted, and only spermatogonia and somatic cells are present in about 20% of the seminiferous tubules of *Stra8*^(^^−^^/^^−^^)^ mice^54^. In addition, our GO analysis revealed that numerous DEGs, e.g., *Dync1h1, Dnah2, Kif4, Kif5b, Chst11*, and *Med12*, were involved in the microtubule-based movement and post-anal tail morphogenesis. These genes have been shown to affect the maturation of male gametes^55–58^. In this study, we found that the percentages of abnormal sperm with round-headed spermatozoa, cytoplasmic droplet, and abnormal sperm tail were increased in P63^(+/^^−^^)^ mice, which indicates that P63 plays an important role in the maturation of male gametes in adult mice.

As P53, P63, and P73 are highly homologous in sequence structure, we compared the DEGs in p63^(+/^^−^^)^ mice with the targeting genes of P53 and P73 to determine whether the DEGs are uniquely regulated by P63. Among the 22 DEGs of P63, it has been shown that *Pomc*^59,60^, *Notch2*^61^, *Igfbp3*^62^, *Jun*^63^, *Csf1*^64^, and *Atm*^65^ are the targeting genes of P53, while *Igfbp3*^66^ and *Jun*^67^ are the targets of P73. *Hes5*^68^, *Igfbp*3^69^, *Jun*^70^, and *Atm*^71^ have been demonstrated to be the target genes of P63. Notably, we identified *Pou5f2*, *Pgk1*, *Prlr*, and *Dll1* as the target genes of P63 by ChIP-qPCR.

In summary, we have utilized P63^(+/^^−^^)^ mice to identify the role and transcriptional regulation of P63 in mediating the apoptosis and adult mouse spermatogenesis. We found that P63^(+/^^−^^)^ mutation resulted in the abnormalities of male germ cells and significant increase of apoptosis in male germ cells. Notably, we have uncovered a number of genes that might be associated with regulating apoptosis and three stages of spermatogenesis. This study thus provides new insights into the death of male germ cells and molecular mechanisms underlying mammalian spermatogenesis. This study could offer new targets for the diagnosis and therapy of male infertility.

## Materials and Methods

### Experimental animals

All mice were fed in the animal center at Renji Hospital, School of Medicine Shanghai Jiao Tong University with controlled temperature and humidity. This study was approved by the Institutional Ethical Review Committee of Ren Ji Hospital, School of Medicine, Shanghai Jiao Tong University. All experimental protocols were performed according to relevant guidelines and regulations of the Institutional Ethical Review Committee of Ren Ji Hospital. The *P63*^Brdm2^ mice were a kind gift from the laboratory of Dr. Weiqiang Gao (Ren Ji Hospital, School of Medicine, Shanghai Jiao Tong University)^7,72^. P63^(+/^^−^^)^ male mice were crossed with wild-type female mice to generate the offspring of P63^(+/^^−^^)^ mice. The genotype of P63^(+/^^−^^)^ mice was determined by PCR analysis using the primer sequences and procedures provided by the Jackson Laboratory (https://www2.jax.org/protocolsdb/f?p=116:5:0::NO:5:P5_MASTER_PROTOCOL_ID,P5_JRS_CODE:5248,003568). For P63^(+/^^−^^)^ genotype, the *p63*^Brdm2^ fragments were amplified by using the following primers: 5′-GTGTTGGCAAGGATTCTGAGACC-3′ (forward), 5′-GGAAGACAATAGCAGGCATGCTG-3′ (reverse). For fecundity assessment, wild type and P63^(+/^^−^^)^ males at 2–8 months old were separately mated with wild-type females for 6 months, and wild type and P63^(+/^^−^^)^ males at around 12 months old were separately mated with wild-type females for 2 months.

### Sperm motility analysis of P63^(+/^^−^^)^ mice and wild type

The sperm motility assay of P63^(+/^^−^^)^ mice and wild-type mice was performed in terms of the method as previously described^73^. In total, 6.3 ± 1.6 × 10^6^ sperm (*n* = 3) from wild-type mice and 5.2 ± 0.9 × 10^6^ sperm (*n* = 3) from P63^(+/^^−^^)^ mice were used to assess the sperm motility and progressive motility. After being euthanized by cervical dislocation, caudal epididymis was immediately excised from the mice. The epididymis was placed into a glass tube containing 1 ml Enriched Krebs-Ringer Bicarbonate (EKRB) medium pre-warmed to 37 °C. The caudal epididymis was cut into small fragments with scissors, and spermatozoa were released into the medium. After 5 min, sperm suspension was put into a Sperm Analysis Chamber (Hamilton Thorne Research, Beverly, USA) and analyzed with a computer-assisted semen analysis (CASA) by the HTM-TOX IVOS sperm motility analyzer (Animal Motility, version 12.3 A, Hamilton Thorne Research). The instrument settings used for the analysis were as follows: temperature, 37 °C; minimum cell size, five pixels; minimum contrast, 50; minimum static contrast, 25; low average path velocity cutoff, 20.0; low straight-line velocity cutoff, 30.0; threshold straightness, 50%; static head size, 0.3–1.95; static head intensity, 0.5–1.3; and magnification, 0.89. Thirty frames were acquired at a frame rate of 60 Hz, and the playback feature was set during the analysis to check the accuracy. The data of the sperm motility were obtained from three independent experiments.

### Transmission electron microscopic (TEM) assay of sperm of P63^(+/^^−^^)^ mice and wild-type mice

The sperms from caudal epididymis of P63^(+/^^−^^)^ mice and wild-type mice were obtained as described above. After being fixed with 2.5% glutaraldehyde in 0.1 M phosphate buffer (pH = 7.4), the ultrastructure of sperm was examined by TEM assay according to the method as previously described^74^. Briefly, the sperm was fixed in 1% osmium tetroxide solution for 2 h, followed by dehydration in a graded series of acetone, and embedded in epoxy resin. Ultrathin sections were sectioned and stained with uranyl acetate and lead citrate. The sections of sperm were examined under a transmission electron microscope (HITACHI, H-7650, Japan).

### Hematoxylin-eosin staining of mouse testes and sperm from P63^(+/^^−^^)^ mice and wild-type mice

P63^(+/^^−^^)^ mice and wild-type mice were killed by cervical dislocation, and testes were immediately fixed in 4% formaldehyde (PFA) overnight. The testis tissues were dehydrated in 30% sugar solution for 24 h at 4 °C, and they were embedded in optimum cutting temperature compound (OTC) and sectioned at 8 μm thicknesses. Testis sections and sperm were stained with hematoxylin-eosin (H&E) pursuant to the method as previously describe^75^. In brief, the testis sections and sperm from P63^(+/^^−^^)^ mice and wild-type mice were obtained and fixed in 4% PFA for 10–15 min, and they were stained with hematoxylin for 15 min and followed by eosin for about 2 min at room temperature. H&E staining of testes and sperm was observed under a phase-contrast microscope, and the percentage of abnormal sperm was counted out of 500 total cells from three independent experiments.

### Isolation of male germ cells from P63^(+/^^−^^)^ and wild-type mice using two-step enzymatic digestion and the differential plating

Two-step enzymatic digestion and the differential plating were utilized to isolate male germ cells from mouse testes of P63^(+/^^−^^)^ and wild-type mice according to the protocol described previously^76^. The testicular tissues from P63^(+/^^−^^)^ and wild-type mice were washed three times with Dulbecco modified Eagle medium (DMEM) (Gibco) containing 2% penicillin and streptomycin (Gibco). The testis tissues were minced by scissors to become a semi-liquid state and then incubated with 10 ml DMEM containing 2 mg/mL type IV Collagenase (Gibco) and 10 μg/ml DNase I (Sigma, Saint Louis, USA) in a shaking water bath at 34 °C at 100 r.p.m. for 10 min. The enzymatic digestion of testis tissues was stopped by 10 ml DMEM containing 10% FBS whenever there were only seminiferous tubules. After 5 min of sedimentation, the seminiferous tubules were incubated with 10 ml DMEM including 2.5 mg/mL type IV collagenase, 2 mg/ml Hyaluronidase (Sigma), 2 mg/ml Trypsin (Sigma) and 10 μg/ml DNase I in oscillating water bath at 34 °C, 100 r.p.m. for 10–15 min. After centrifugation at 300×*g* for 5 min, the supernatant was removed. The sediment was re-suspended in DMEM/F-12 (Gibco) supplemented with 10% FBS, and cell suspension was filtered through a 40 µm nylon mesh to remove cell aggregates. The cell mixture was seeded in a 10 cm dish pre-coated with gelatin, and they were cultured at 34 °C in 5% CO_2_ for 24 h. Somatic cells, including sertoli cells, leydig cells, and myoid cells, were attached to culture dishes, and male germ cells remained in suspension.

### Isolation of spermatogonia, pachytene spermatocytes, and round spermatids from P63^(+/^^−^^)^ mice and wild-type mice by STA-PUT velocity sedimentation

The STA-PUT method, using a linear bovine serum albumin (BSA) (Sigma) gradient and sedimentation, was utilized to separate spermatogonia, pachytene spermatocytes, and round spermatids from P63^(+/^^−^^)^ mice and wild-type mice in terms of the size, mass and gravity of heterogeneous cells. Male germ cells were obtained using two-step enzymatic digestion and followed by the differential plating from P63^(+/^^−^^)^ mice and wild-type mice at 2 months old, and spermatogonia, pachytene spermatocytes, and round spermatids were isolated by STA-PUT velocity sedimentation from ~5 × 10^7^ male germ cells according to the protocol as we described previously^11^. In terms of cell diameter and morphology under microscope, 5–25 fraction collections were chosen as the pachytene spermatocytes, and 35–50 fraction collections were selected as spermatogonia. In addition, 60–75 fraction collections were regarded as round spermatids. An aliquot of each cell fraction was examined carefully under a phase-contrast microscope to assess cellular integrity and identify of each cell type. Trypan blue staining was performed to assess the viability of the freshly isolated germ cells. Briefly, the freshly isolated germ cells were stained with 0.4% trypan blue for 3–5 min, and the viability of these cells was assessed by the percentage of the cells excluding trypan blue and total cells counted. RT-PCR, immunocytochemistry and meiotic spread assays were used to determine the phenotype and purity of the isolated spermatogonia, pachytene spermatocytes and round spermatids. The mRNA was extracted from spermatogonia, pachytene spermatocytes, and round spermatids of P63^(+/^^−^^)^ and wild-type mice for RNA sequencing.

### PCR for *Trp63*^Brdm2^ and *P63* allele in P63^(+/^^−^^)^ and wild-type mice

Genomic DNA was extracted from tails of P63^(+/^^−^^)^ and wild-type mice using DNA extraction kit (Biomed, PC3201, China). The primer sequences of *P63* and *Actb* were designed and listed in Table S1. The PCR reactions started at 94 °C for 2 min and were performed pursuant to the following conditions: denaturation at 94 °C for 30 s, annealing at 60 °C for 45 s, and elongation at 72 °C for 45 s, for 26 cycles. The PCR samples were incubated for an additional 5 min at 72 °C. PCR products were separated by electrophoresis with 2% agarose gel and they were visualized with ethidium bromide.

### RNA extraction and reverse transcription (RT) and real-time quantitative PCR

Total RNA was extracted from spermatogonia, pachytene spermatocytes, and round spermatids of P63^(+/^^−^^)^ mice and wild-type mice using Trizol (Takara, Kusatsu, Japan). The concentrations of total RNA were measured by Nanodrop (Thermo), and RIN (RNA integrity number) of >7 of total RNA was used to ensure good quality. The First Strand cDNA Synthesis Kit (Thermo Scientific, USA) was used to conduct reverse transcription (RT) of total RNA, and PCR of the cDNA was carried out according to the protocol as we described previously^77^. The primer sequences of the chosen genes for RT-PCR were designed and listed in Table S1. The PCR reactions started at 94 °C for 2 min and were performed in terms of the following conditions: denaturation at 94 °C for 30 s, annealing at 55–60 °C for 45 s as listed in Table S1, and elongation at 72 °C for 45 s, for 35 cycles. The PCR samples were incubated for an additional 5 min at 72 °C. RNA without RT (RT−) but with PCR served as a negative control. PCR products were separated by electrophoresis with 2% agarose gel and they were visualized with ethidium bromide.

Quantitative real-time PCR reactions were conducted using Power SYBR Green PCR Master Mix (Applied Biosystems, Woolston Warrington, UK) and a 7500 fast real-time PCR system (Applied Biosystems, Carlsbad, CA, USA). The primer sequences of the chosen genes for real-time PCR were designed and listed in Table S1. The comparative CT (Threshold Cycle) method was used to quantify the PCR products. The CT value of gene was normalized against the threshold value of mice housekeeping gene *Actb* [ΔCT = CT_(Gene)_−CT_(*Actb*)_], and the relative expression of gene in P63 mutation mice to the wild-type mice was calculated by formula 2^−ΔΔCT^[ΔΔCT = ΔCT_(P63 mutation)_−ΔCT _(wild type)_].

### Immunocytochemistry

For immunocytochemistry, freshly isolated spermatogonia and round spermatids from P63^(+/^^−^^)^ and wild-type mice were fixed with 4% PFA for 15–30 min, and they were washed three times with cold PBS (Medicago, Uppsala, Sweden) and permeabilized with 0.4% Triton X-100 (Sigma) for 5–15 min. After extensive washes with PBS, these cells were blocked in 5% BSA for 1 h at room temperature and followed by incubation with primary antibodies, including GPR125 (Abcam, ab51705, 1:200), GFRA1 (Abcam, ab8026, 1:200), UCHL1 (AbDSerotec, MCA4750, 1:200), THY1 (Abcam, ab133350 1:200), PRM1 (Santa Cruz Biotechnology, sc-30173, 1:200) overnight at 4 °C. The specificity of these antibodies was evaluated previously and verified to be great^11^. After extensive washes with PBS, the cells were incubated with IgGs conjugated with fluorescein isothiocyanate (FITC) (Sigma) or rhodamine-conjugated IgG (Sigma), at a 1:200 dilution for 1 h at room temperature. Replacement of primary antibodies with isotype IgGs in mouse male germ cells served as negative controls. DAPI (4, 6-diamidino-2-phenylindole) was employed to label the cell nuclei, and the images were captured with a Nikon microscope (Tokyo, Japan).

### Meiotic spread assays

Meiotic spread assays were performed to determine the identity and the purity of the freshly isolated pachytene spermatocytes from P63^(+/^^−^^)^ and wild-type mice testis by STA-PUT velocity sedimentation pursuant to the method described previously^11^. Briefly, cells were lysed by a hypotonic solution and spread evenly over slides layered with 1% PFA and 0.15% Triton X-100. Slides were dried for 24 h at room temperature in a humid chamber. The cells were treated with 0.04% photoflo for 5 min and blocked with 4% goat serum. The cells were incubated with primary antibodies, including SCP3 (Abcam, ab15093, 1:100), MLH1 (BD Pharmingen, 551092, 1:50) and CREST (Immunovision, HCT-0100, 1:80), overnight at 37 °C in a humid chamber. Donkey anti-rabbit IgG(H + L) (Alexa Fluor 555, Molecular Probes, A31572, 1:250), Alexa Fluor 488 goat anti-mouse IgG1(γ1) (AF488, Molecular Probes, A-21121, 1:120) and AMCA-conjugated affiniPure donkey anti-human IgG(H + L) (Jackson ImmunoResearch, 709–155–149, 1:200) were used as the secondary antibodies and incubated for 90 min at 37 °C. After washing three times with cold TBS, the cells were observed with a fluorescence microscope (Nikon, Tokyo, Japan).

### PNA-FITC staining

Acrosome integrity of round spermatids from P63^(+/^^−^^)^ and wild-type mice was detected by PNA-FITC staining. Briefly, the isolated round spermatids were fixed in 4% PFA for 10–15 min. After washing with PBS three times per 5 min, the cells were incubated with PNA-FITC (Invitrogen, 1:300) for 30 min at room temperature. DAPI (4, 6-diamidino-2-phenylindole) was employed to label the cell nuclei, and the images were captured with a Nikon microscope.

### Immunohistochemistry

Testis sections of P63^(+/^^−^^)^ mice and wild-type mice were deparaffinized by xylene three times, and hydrated with a series of graded alcohol and treated with 3% H_2_O_2_ (Boster Biological Technology, Guangzhou, China) for 10 min at room temperature to block the endogenous peroxidase activity. After blocking with 5% BSA for 1 h at room temperature, the sections were incubated with primary antibody against P63 (Abcam, ab735, 1:200) overnight at 4 °C. After extensive washes with PBS, the sections were incubated with HRP conjugated second antibody for 1 h at room temperature. After extensive washing with PBS, DAB (3, 3-diaminobenzidine) (Zhongshanjinqiao Biotechnology, Beijing, China) was used to label the P63 protein. Finally, the sections were stained with hematoxylin and observed under a Nikon microscope.

### TUNEL assay

The apoptosis percentages of male germ cells in testis sections of wild-type mice and P63^(+/^^−^^)^ mice were detected using the TUNEL Apoptosis Detection Kit pursuant to the manufacturer’s instruction (YEASEN, China). Testis sections of wild type and P63^(+/^^−^^)^ mice were deparaffinized by xylene for three times, and they were hydrated with a series of graded alcohol. After being treated with 20 μg/ml proteinase K (YEASEN, China) for 20 min at room temperature, the sections were incubated with FITC-12-dUTP labeling/TdT enzyme buffer for 1 h in dark, and PBS without TdT enzyme was used as the negative control. Propidium iodide (PI) was used to label the nuclei, and images were observed under a Nikon microscope. The percentages of TUNEL-positive cells were counted in at least 500 cells, and three independent experiments were performed.

### Western blots

Male germ cells from wild-type mice and P63^(+/^^−^^)^ mice were lysed with RIPA buffer (BiotechWell, Shanghai, China) for 30 min on ice. Cell lysates were cleared by centrifugation at 12,000×*g* for 15 min at 4 °C, and the protein concentrations were measured by BCA kit (DingGuo ChangShengBiotech, Beijing, China). In total, 30 mg of cell lysate from each sample were separated using 10% SDS-PAGE (Bio-Rad Laboratories) and transferred to nitrocellulose membranes for 2 h at room temperature. The membranes were blocked using 5% nonfat dry milk in TBS-T for 1 h at room temperature. After washing with TBS-T, the membranes were incubated with antibodies against P63 (Abcam, ab735, 1:500) and ACTB (Protein tech, catalog no: HRP-60008, dilution: 1:5000) overnight at 4 °C. After extensive washes, the membranes were incubated with horseradish peroxidase-conjugated immunoglobulin G (IgG) (Santa Cruz Biotechnology) at a 1:2000 dilution for 1 h at room temperature. The membranes were detected by chemiluminescence (Chemi-Doc XRS, Bio-Rad, Hercules, CA, USA), and densitometric analyzes were processed with Adobe Photoshop 8.0. The relative level of P63 protein was normalized to the expression of ACTB.

### Annexin V/propidium iodide (PI) staining and flow cytometry

The apoptosis percentages of male germ cells of wild type and P63^(+/^^−^^)^ mice were detected using the Annexin V-FITC/PI kit by flow cytometry pursuant to the manufacturer’s instruction (Biolegend, London, UK). Male germ cells were obtained from P63^(+/^^−^^)^ mice and wild-type mice using two-step enzyme and followed by the differential plating and culture for 24 h. It was feasible to identify and quantify apoptotic cells by conjugating FITC to Annexin V using flow cytometry. The cells were simultaneously stained with Annexin V-FITC (green fluorescence) and the non-vital dye PI (red fluorescence), which allowed the discrimination of intact cells (FITC^−^PI^−^), early apoptotic (FITC^+^PI^−^) and late apoptotic or necrotic cells (FITC^+^PI^+^). The procedure was carried out according to the protocol as described previously^77^.

### RNA sequencing

Total RNAs were extracted from spermatogonia, pachytene spermatocytes, and round spermatids of P63^(+/^^−^^)^ mice and wild-type mice with Trizol reagent (Invitrogen, Carlsbad, CA, USA). The quantity and purity of total RNA were analyzed with Agilent 2100 Bioanalyzer (Agilent RNA 6000 Nano Kit) with RIN >7.0. The cDNA libraries were used for sequencing on an Illumina HiSeq 2500 instrument. RNA sequencing libraries were established for two RNA sequencing samples (three mice per sample) at Guangzhou RiboBio Co., Ltd (Guangzhou, China) using the NEBNext Ultra RNA Library Prep Kit (Illumina, San Diego, USA), and the procedures and standards were performed in terms of the manufacture’s manual. A computational pipeline was employed to process RNA-sequencing data. Clean reads were obtained after the raw reads were filtered with adaptor skewer^78^, and sequencing data were mapped to mouse genome mm10 RefSeq with Tophat version 2.0.13 with default parameters^79^. Gfold version 1.1.2 was used to produce biologically meaningful rankings of differentially expressed genes (DEGs)^80^. Subsequently, those DEGs were assessed by Audics using RPKM^81^, and the significantly DEGs were selected according to the following criteria: *p* < 0.05 and fold change ≥2.

### Chromatin immunoprecipitation-qPCR assay

Chromatin immunoprecipitation (ChIP) assay was performed according to the manufacturer’s instruction (Beyotime Biotechnology, P2078, China). Human spermatogonia, pachytene spermatocytes and round spermatids were isolated from wild-type mice using STA-PUT, and they were cross-linked with 1% formaldehyde for 10 min at 37˚C. After terminating with 125 mM glycine solution and washing with PBS, the cells were lysed with lysis buffer containing 1 mM PMSF for 10 min in ice-bath. Chromatin solutions were sonicated to 300–700 bp DNA fragments using sonicator. After being diluted with chip dilution buffer, 20 μl samples were taken out for input control. The sonicated DNA fragments were incubated with anti-IgG (Sigma) or anti-P63 antibody (Abcam) overnight at 4˚C. Immunocomplexes were washed with low salt buffer, high-salt buffer, and LiCl buffer, and they were eluted with elution buffer (1% SDS, 0.1 M NaHCO_3_). Cross-links were reversed at 65 °C for 4 h. DNA was purified using PCR purification Kit (Beyotime biotechnology, P2078, China). Enrichment was measured using qPCR, and the primers of genes were listed in Supplemental Table S1. Fold enrichment was calculated using the comparative threshold cycle method using the formula 2^−ΔΔCT^ as mentioned above.

### Statistical analysis

All data were presented as mean ± SD and analyzed by Student’s *t*-test or one way ANOVA with the appropriate post hoc tests using Prism (version 5, GraphPad Software). Normality and homogeneity of variances were checked prior to conduct Student’s *t*-test or one way ANOVA, and *P* < 0.05 was considered statistically significant.

## Supplementary information

8 Supplemental Tables & 3 Supplementa Figure 3
