# Supplementary material for: Transcriptional regulation of P63 on the apoptosis of male germ cells and three stages of spermatogenesis in mice
Source: Cell Death Dis. 2018 Jan 23;9(2):76. doi: 10.1038/s41419-017-0046-z (PMC5833356; doi:10.1038/s41419-017-0046-z)
Supplement: Supplementary file 1 — 8 Supplemental Tables & 3 Supplementa Figure 3 [file 41419_2017_46_MOESM1_ESM.docx]

**Supplemental Information**

**Transcriptional regulation of P63 on the apoptosis** **of male germ cells and three stages of spermatogenesis in mice**

Hong Wang^1*^, Qingqing Yuan^1*^, Minghui Niu^1*^, Wenhui Zhang^1^, Liping Wen^1^, Hongyong Fu^1^, Fan Zhou^1^ & Zuping He^1, 2, 3, 4#^

**Supplemental Tables 1-8**

**Supplemental Figures 1-3**

**Supplemental Tables**

**Table S1. Primer sequences of genes used for quantitative real-time PCR, RT-PCR, PCR, and ChIP-qPCR**

| **Genes** | **Primer sequences** | **Product size (bp)** | **Tm (℃)** |
| --- | --- | --- | --- |
| **Primer sequences used for quantitative real-time PCR** | | | |
| *Csf1* | F: 5’- GGCTTGGCTTGGGATGATTCT-3’ | 126 | 61 |
|  | R: 5’- GAGGGTCTGGCAGGTACTC-3’ |  |  |
| *Junb* | F: 5’- CTATCGGGGTCTCAAGGGTC-3’ | 147 | 59 |
|  | R: 5’- CTGTTGGGGACGATCAAGC-3’ |  |  |
| *Notch2* | F: 5’- ATGTGGACGAGTGTCTGTTGC-3’ | 146 | 59 |
|  | R: 5’- GGAAGCATAGGCACAGTCATC-3’ |  |  |
| [*Zbtb16*](https://www.ncbi.nlm.nih.gov/gene/235320) | F: 5’- CTGCGGAAAACGGTTCCTG-3’ | 150 | 60 |
|  | R: 5’- GTGCCAGTATGGGTCTGTCT-3’ |  |  |
| *Stra8* | F: 5’- ACCCTGGTAGGGCTCTTCAA-3’ | 192 | 60 |
|  | R: 5’- GACCTCCTCTAAGCTGTTGGG-3’ |  |  |
| *Bmpr2* | F: 5’- CCTGACACAACACCACTCAGT-3’ | 103 | 60 |
|  | R: 5’- AGGCGACTATCAAAACAGCTAAC-3’ |  |  |
| *Fmr1* | F: 5’- AGGTGCCAGAAGATTTACGACA-3’ | 147 | 60 |
|  | R: 5’- CTCGCTTTGAGGTGACTTCATT-3’ |  |  |
| *Igfbp3* | F: 5’- CACACCGAGTGACCGATTCC-3’ | 121 | 61 |
|  | R: 5’- GTGTCTGTGCTTTGAGACTCAT-3’ |  |  |
| *Pgk1* | F: 5’- ATGTCGCTTTCCAACAAGCTG-3’ | 164 | 60 |
|  | R: 5’- GCTCCATTGTCCAAGCAGAAT-3’ |  |  |
| *Cyp17a1* | F: 5’- AGTCAAAGACACCTAATGCCAAG-3’ | 83 | 60.5 |
|  | R: 5’- ACGTCTGGGGAGAAACGGT-3’ |  |  |
| *Pou5f2* | F: 5’- GGTCTTCAAACGTCTTCCCTC-3’ | 118 | 61 |
|  | R: 5’- CCCATACTTGGTCGCACCAT-3’ |  |  |
| *Jun* | F: 5’- TTCCTCCAGTCCGAGAGCG-3’ | 133 | 62 |
|  | R: 5’- TGAGAAGGTCCGAGTTCTTGG-3’ |  |  |
| *Atm* | F: 5’- CCAGCTTTTTGATGCAGATACCA-3’ | 119 | 60.5 |
|  | R: 5’- CTTCCCAGCCTACGTCTATTTTC-3’ |  |  |
| *Dll1* | F: 5’- CCCATCCGATTCCCCTTCG-3’ | 100 | 61.5 |
|  | R: 5’- GGTTTTCTGTTGCGAGGTCATC-3’ |  |  |
| *Tgfa* | F: 5’- CACTCTGGGTACGTGGGTG-3’ | 136 | 61 |
|  | R: 5’-CACAGGTGATAATGAGGACAGC-3’ |  |  |
| *Prlr* | F: 5’- CTGCACTTGCTTACATGCTGC-3’ | 94 | 60.5 |
|  | R: 5’- GGGGAACGACATTTGTGGATTTC-3’ |  |  |
| *Pomc* | F: 5’- ATGCCGAGATTCTGCTACAGT-3’ | 75 | 60 |
|  | R: 5’- CCACACATCTATGGAGGTCTGAA-3’ |  |  |
| *Pld6* | F: 5’- CAGCGAGAGTTCACTGAGCC-3’ | 166 | 61 |
|  | R: 5’- GGCCATGTAGTCGCAGTCA-3’ |  |  |
| *Cdkn2d* | F: 5’- CTGGAAGAAGTCTGCGTCGG-3’ | 125 | 62 |
|  | R: 5’- GTCTTGCCAAAGCGGTTCAG-3’ |  |  |
| *Dazl* | F: 5’- TCTGCCACAACTTCTGAGGC-3’ | 168 | 60.5 |
|  | R: 5’- CCTGATTTCGGTTTCATCCATCC-3’ |  |  |
| *Hes5* | F: 5’- AGTCCCAAGGAGAAAAACCGA-3’ | 183 | 61 |
|  | R: 5’- GCTGTGTTTCAGGTAGCTGAC-3’ |  |  |
| *Insl3* | F: 5’- CCTTGGAGGATCCCTAGTTTATTC-3’ | 105 | 58 |
|  | R: 5’- GGACACAGGGAGGAGGT-3’ |  |  |
| *Actb* | F: 5’- GTGCTATGTTGCTCTAGACTTCG-3’ | 174 | 59 |
|  | R: 5’- ATGCCACAGGATTCCATACC-3’ |  |  |
| **Primer sequences used for RT-PCR and PCR** | | | |
| *Gfra1* | F: 5’-TGCGTATCTACTGGAGCATGT-3’ | 171 | 61.5 |
|  | R: 5’-CATCGAGGCAGTTGTTCCCTT-3’ |  |  |
| *Gpr125* | F: 5’-CGGTCACCCTGATTTTAAGCA-3’ | 124 | 61.5 |
|  | R: 5’-GCACCTGGGGCTATCCTACTAA-3’ |  |  |
| [*Zbtb16*](https://www.ncbi.nlm.nih.gov/gene/235320) | F: 5’- CTGCGGAAAACGGTTCCTG-3’ | 150 | 60 |
|  | R: 5’- GTGCCAGTATGGGTCTGTCT-3’ |  |  |
| *Thy1* | F: 5’-GCTCTCAGTCTTGCAGGTGTC-3’ | 79 | 61.5 |
|  | R: 5’-CAGGCGAAGGTTTTGGTTCA-3’ |  |  |
| *Scp3* | F: 5’-AGCCAGTAACCAGAAAATTGAGC-3’ | 106 | 61 |
|  | R: 5’-CCACTGCTGCAACACATTCATA-3’ |  |  |
| *Crest* | F: 5’-GAGCAAGAACAGTCATCAGCG-3’ | 195 | 61 |
|  | R: 5’-AGGTGATTCCAGTAAAGATGCCT-3’ |  |  |
| *Mlh1* | F: 5’-CAAGCCCCTCCTAAACCCTG-3’ | 237 | 62 |
|  | F: 5’-GTCCACGGTTGTGGCATTG-3’ |  |  |
| *Acrosin* | F: 5’-ACTGGAGACTGGTTTTCGGAG-3’ | 146 | 60.5 |
|  | R: 5’-CAAGAGCGCAATGTCATTTCC-3’ |  |  |
| *Tnp1* | F: 5’-ACCAGCCGCAAGCTAAAGAC-3’ | 120 | 61 |
|  | R: 5’-TTTCCTACTTTTCAGGACGCTC-3’ |  |  |
| *Prm1* | F: 5’-CCGTCGCAGACGAAGATGTC-3’ | 96 | 61.5 |
|  | R: 5’-CACCTTATGGTGTATGAGCGG-3’ |  |  |
| *P63* | F: 5’-TCGATGCAGTCTCAGTCTTCAT | 123 | 60 |
|  | R: 5’-TGCCCTCAGGCATGGTGGT |  |  |
| *Actb* | F: 5’- CTCCCTGGAGAAGAGCTATGA-3’ | 266 | 62 |
|  | R: 5’- TAGGAGCCAGAGCAGTAATCT-3’ |  |  |
| **Primer sequences used for ChIP-qPCR** | | | |
| *Pomc* | F: 5’-RTAGCTTGTGCTGTGCTCTCTA-3’ | 145 | 60 |
|  | R: 5’-TGAGCCAACACTAACAGCTCTTA-3’ |  |  |
| *Pou5f2* | F: 5’-AGGACTGACTAAGTTACCTCAGT-3’ | 101 | 59 |
|  | R: 5’-TTCTGAACAGCCACTAGCCT-3’ |  |  |
| *Pgk1* | F: 5’-ACACATTCCACATCCACCGGT-3’ | 156 | 60 |
|  | R: 5’-AGACTAGTGAGACGTGCTACTT-3’ |  |  |
| *Prlr* | F: 5’-TCTCAATCTGGCAGGATCGAT-3’ | 238 | 60 |
|  | R: 5’-AGACAACAGTGTAGAGAGTTCA-3’ |  |  |
| *Jun* | F: 5’-AGCCAGCTTCAGTCACTCAGA-3’ | 268 | 60 |
|  | R: 5’-TGCATTGTGGGCTGACGTCTT-3’ |  |  |
| *Dll1* | F: 5’-TGACAGCTCTCGCCAGATT-3’ | 222 | 61 |
|  | R: 5’-TCTTCCTCAAGAATTCAGCCAA-3’ |  |  |
| Control primer | F: 5’- GTGCTATGTTGCTCTAGACTTCG-3’ | 174 | 59 |
|  | R: 5’- ATGCCACAGGATTCCATACC-3’ |  |  |

**Table S2.** **The reads and gene numbers in spermatogonia, pachytene spermatocytes and round spermatids of P63^(+/-)^ mice and wild type mice**

| **Cell Types** | **Total reads** | **Gene numbers** |
| --- | --- | --- |
| Spermatogonia of wild type mice | 16,497,158 | 27,100 |
| Spermatogonia of P63^(+/-)^ mice | 18,381,820 | 26,977 |
| Pachytene spermatocytes wild type | 18,300,145 | 27,714 |
| Pachytene spermatocytes of P63^(+/-)^ mice | 18,495,358 | 27,686 |
| Round spermatids wild type | 16,933,076 | 26,283 |
| Round spermatid of P63^(+/-)^ mice | 16,828,312 | 23,308 |

**Table S3.** **The GO analysis of the DEGs in spermatogonia between P63^(+/-)^ mice and wild type mice**

| **GO** | **Gene numbers** | **Gene names** |
| --- | --- | --- |
| [Spermatogenesis](http://www.ebi.ac.uk/QuickGO/GTerm?id=GO:0007283) | 22 | *Axl, Bax , Brip1, Dmc1, Prdm9, Rad21l, Atrx, Apob, Ctsl, Cldn11, Dazl, Dmrt1, Gal3st1, Hist1h1a, Igf2r, Kit, Pld6, Sohlh2, Sgpl1, Stra8, Trp63, Ubr2* |
| [Negative regulation of apoptotic process](http://www.ebi.ac.uk/QuickGO/GTerm?id=GO:0043066) | 28 | *Axl, Grk5, Xiap, Alb, Aldh2, Chst11, Clu, Cat , Cryab , Ccnd2, Eif2ak3, Fn1, Gpx1, Gnaq, Hspd1, Hif1a, Id1, Insl3, Igf1r, Kitl, Lef1, Map4k4, Ncl, Spp1, Serpinb2, Tex11, Trp63, Tgfa* |
| [Positive regulation of apoptotic process](http://www.ebi.ac.uk/QuickGO/GTerm?id=GO:0043065) | 21 | *Bax , Klf11, Aldh1a1, Anxa5, Aifm1, Atm, Clu, Hspd1, Hif1a, Igf2r, Itgb1, Lrp6, Notch2, Ptgs2, Ripk2, Smpd1, Trp73, Trpm7, Tnfrsf12a , Zbtb16, Zfp346* |
| [Germ cell development](http://www.ebi.ac.uk/QuickGO/GTerm?id=GO:0007281) | 7 | *Bax , Cxcl12, Dazl, Kitl, Kit, Lin28a, Msh2* |
| [Positive regulation of MAPK cascade](http://www.ebi.ac.uk/QuickGO/GTerm?id=GO:0043410) | 9 | *C1qtnf1, Cd36, Agt, Igf1r, Itgav , Itgb1, Kit, Psap, Ryk* |
| [Positive regulation of cell migration](http://www.ebi.ac.uk/QuickGO/GTerm?id=GO:0030335) | 14 | *Ets1，Cxcl12，Cpne3, Cpeb1, Fn1, Igf1r, Itgav, Itgb1, Kit, Lamb1, Lef1 , Mcam, Ret, Rras2* |
| [Wnt signaling pathway](http://www.ebi.ac.uk/QuickGO/GTerm?id=GO:0016055) | 14 | *Grk5, Ldb1, Mark2, Xiap, Cpe, Csnk1e, Fermt2, Lrp6, Lef1, Ryk, Spin1, Tnks2, Tle4, Zbtb33* |
| [Germ cell migration](http://www.ebi.ac.uk/QuickGO/GTerm?id=GO:0008354) | 4 | *Dmrt1, Itgb1, Kit, Cxcl12* |
| [Cell cycle](http://www.ebi.ac.uk/QuickGO/GTerm?id=GO:0007049) | 25 | *Arl8a, Cd2ap, Dmc1, E2f2, E2f6, Rassf4, Taf1, Tspyl2, Appl1, Atm, Ccnb3, Ccnd2, Cdkn2d, Esco2, Eif2ak4, Fam83d, Lzts1, Mcm2, Snx18, Msh2, Spin1, Smc1a, Trp63, Trp73, Usp22* |
| [Cell cycle arrest](http://www.ebi.ac.uk/QuickGO/GTerm?id=GO:0007050) | 7 | *Atm, Cdkn2d, Eif2ak4, Inhba, Msh2, Notch2, Trp73* |
| [Cell differentiation](http://www.ebi.ac.uk/QuickGO/GTerm?id=GO:0030154) | 36 | *Axl, Cebpb, Ets1, Elavl3, Epha2, Jak1, Mark2, Agrn, Apaf1, Col13a1, Dazl, Dmrt1, Etv5, Eif2ak4, Grb2, Hes5, Hif1a, Ildr2, Itgav, Kit, Mdk, Myrf, Notch2, Peg10, Pld6, Pkdcc, Rarres2, Sema6d, Sohlh2, Srms, Stra8, Tll1, Tcf3, Trp63, Tnfrsf12a, Zbtb7b* |
| [Positive regulation of cell proliferation](http://www.ebi.ac.uk/QuickGO/GTerm?id=GO:0008284) | 25 | *Ets1, Grk5, Taf1, Agt, Cxcl12, Clu, F2, Ccnd2, Ddr2, Fn1, Hes5, Hif1a, Insl3, Itgav, Itgb1, Kitl, Kit, Lef1, Kdm4c, Mecp2, Notch2, Ptgs2, Stx3, Tns3, Tgfa* |
| [Negative regulation of cell proliferation](http://www.ebi.ac.uk/QuickGO/GTerm?id=GO:0008285) | 17 | *Bax, Brip1, Klf11, Nrk, Agt, Asph, Cdkn2d, Cpeb1, Gja1, Inhba, Insl3, Itgb1, Kmt2a, Notch2, Ptgs2, Ski, Zbtb16* |
| [Apoptotic cell clearance](http://www.ebi.ac.uk/QuickGO/GTerm?id=GO:0043277) | 4 | *Axl, Cd36, Lrp1, Itgav* |
| [Male germ-line stem cell asymmetric division](http://www.ebi.ac.uk/QuickGO/GTerm?id=GO:0048133) | *3* | *Stra8, Zbtb16, Etv5* |

**Table S4.** **The GO analysis of the DEGs in pachytene spermatocytes between P63^(+/-)^ mice and wild type mice**

| **GO** | **Gene numbers** | **Gene names** |
| --- | --- | --- |
| [Steroid biosynthetic process](http://www.ebi.ac.uk/QuickGO/GTerm?id=GO:0006694) | 6 | *Cyp11a1, Cyp17a1, Hsd3b1, Hsd3b6, Hmgcs2, Hsd17b3* |
| [Oxidation-reduction process](http://www.ebi.ac.uk/QuickGO/GTerm?id=GO:0055114) | 8 | *Adh1, Cyp11a1, Cyp17a1, Egln1, Hsd3b1, Hsd3b6, Egln3, Hsd17b3* |
| [Glucocorticoid biosynthetic process](http://www.ebi.ac.uk/QuickGO/GTerm?id=GO:0006704) | *2* | *Cyp11a1, Cyp17a1* |
| [Positive regulation of apoptotic process](http://www.ebi.ac.uk/QuickGO/GTerm?id=GO:0043065) | *4* | *Egln1, Frzb, Igfbp3, Phlda3* |
| [Response to steroid hormone](http://www.ebi.ac.uk/QuickGO/GTerm?id=GO:0048545) | 2 | *Adh1, Paqr5* |
| [Intrinsic apoptotic signaling pathway in response to DNA damage by p53 class mediator](http://www.ebi.ac.uk/QuickGO/GTerm?id=GO:0042771) | 2 | *Phlda3, Trp63* |

**Table S5.** **The GO analysis of the DEGs in round spermatids between P63^(+/-)^ mice and wild type mice**

| **GO** | **Gene numbers** | **Gene names** |
| --- | --- | --- |
| [Positive regulation of cell migration](http://www.ebi.ac.uk/QuickGO/GTerm?id=GO:0030335) | 28 | *Cd274, Ets1, Apc, Bcar1, Csf1, C3ar1, Cyr61, Cpeb1, Dab2, Fam83h, Fn1, Itga5, Kit, Lamb1, Mcam, Mylk, Plau, Pdgfra, Pdgfa, Sema7a, Sema3b，Sema3f，Sema4b， Sema4c， Sema4g， Syne2，Stx4a，Vegfa* |
| [Microtubule-based movement](http://www.ebi.ac.uk/QuickGO/GTerm?id=GO:0007018) | 9 | *Bicd2, Dync1h1, Dnhd1, Dnah17, Dnah2, Dnah8, Kif4, Kif5b* |
| [Apoptotic process](http://www.ebi.ac.uk/QuickGO/GTerm?id=GO:0043066) | 31 | *Bcl2l2, Brat1, Cd27, Cd5l, Ddit4, Ep300, Fasl, Htra2, Madd, Traf2, Birc6, Casr, Dab2ip, Dab2, Gsn, Gapdh, Gadd45b, Hmox1, Hip1, Ing4, Nradd, Pidd1, Peg3, Ppp1r15a, Six1, Sgpl1, Trp53bp2, Trp63, Trp73, Tnfrsf12a, Ube2z* |
| [Positive regulation of apoptotic process](http://www.ebi.ac.uk/QuickGO/GTerm?id=GO:0043065) | 24 | *Fasl, Htra2, Apc, Anxa5, Atm, Cyr61, Dab2ip, Gpld1, Gadd45a, Gadd45b, Hmox1, Ing4, Igf2r, Jun, Lrp6, Nos3, Notch2, Ptprf, Zak, Tfap4, Trp73, Tgfb3, Tgm2, Tnfrsf12a* |
| [Apoptotic cell clearance](http://www.ebi.ac.uk/QuickGO/GTerm?id=GO:0043277) | 4 | *Axl , Lrp1, Rara, Tgm2* |
| [Cell cycle arrest](http://www.ebi.ac.uk/QuickGO/GTerm?id=GO:0007050) | 10 | *Apc, Atm, Foxo4, Gadd45a , Ing4, Msh2, Notch2, Zak, Trp73, Zbtb49* |
| [Post-anal tail morphogenesis](http://www.ebi.ac.uk/QuickGO/GTerm?id=GO:0036342) | 6 | *Akp3, Chst11, Lrp6, Med12, Trp63, Wnt3a* |
| [Aging](http://www.ebi.ac.uk/QuickGO/GTerm?id=GO:0007569) | 22 | *Atp2b1, Fos, Htra2, Scap, Ctnna1, Ccl2, Cryab, Dcn, Dmd, Foxo4, Gsn, Gclc, Igfbp2, Itgb2, Jun, Lrp1, Serping1, Srebf1, Tyms, Tgfb3, Tnfrsf1b* |

**Table S6.** **The pathway analysis of the DEGs in spermatogonia between P63^(+/-)^ mice and wild**

**type mice**

| **Pathway** | **Sequence in biological process** | **Gene names** | **Fold enrichment** | **Percentage of genes in list** | **P value** |
| --- | --- | --- | --- | --- | --- |
| [PPAR signaling pathway](https://david.ncifcrf.gov/kegg.jsp?path=mmu03320$PPAR%20signaling%20pathway&termId=550039783&source=kegg) | 11 | *Cd36, Acsbg1, Acsl4, Apoa1, Apoa5, Cyp4a10, Cyp4a14, Pck1, Slc27a2, Scd1, Scd2* | 4.50 | 1.71 | 1.43x10^-4^ |
| [p53 signaling pathway](https://david.ncifcrf.gov/kegg.jsp?path=mmu04115$p53%20signaling%20pathway&termId=550039807&source=kegg) | 7 | *Bax, Apaf1, Atm, Ccnb3, Ccnd2, Serpine1, Trp73* | 3.42 | 1.09 | 1.59 x 10^-2^ |
| [PI3K-Akt signaling pathway](https://david.ncifcrf.gov/kegg.jsp?path=mmu04151$PI3K-Akt%20signaling%20pathway&termId=550039818&source=kegg) | 18 | *Epha2, Jak1, Rac1, Creb3l2, Ccnd2, Fn1, Grb2, Igf1r, Itgav, Itgb1, Kitl, Kit, Lamc1, Pck1, Prlr, Spp1* | 1.68 | 2.79 | 3.84 x 10^-2^ |
| [cGMP-PKG signaling pathway](https://david.ncifcrf.gov/kegg.jsp?path=mmu04022$cGMP-PKG%20signaling%20pathway&termId=550039795&source=kegg) | 12 | *Atp2b1, Atp1a1, Atp1b2, Rock2, Adcy7, Creb3l2, Cngb1, Gnaq, Mef2b, Mylk3, Slc25a4, Vdac1* | 2.30 | 1.90 | 1.51 x 10^-2^ |

**Table S7.** **The pathway analysis of the DEGs in pachytene spermatocytes between P63^(+/-)^ mice and wild type mice**

| **Pathway** | **Sequence in Biological Process** | **Gene Name** | **Fold Enrichment** | **Percentage of genes in list** | **P Value** |
| --- | --- | --- | --- | --- | --- |
| [Steroid hormone biosynthesis](https://david.ncifcrf.gov/kegg.jsp?path=mmu00140$Steroid%20hormone%20biosynthesis&termId=550039692&source=kegg) | 5 | *Cyp11a1, Cyp17a1, Hsd3b1, Hsd3b6, Hsd17b3* | 4.50 | 12.7 | 5.39 x 10^-4^ |
| [Endocrine and other factor-regulated calcium reabsorption](https://david.ncifcrf.gov/kegg.jsp?path=mmu04961$Endocrine%20and%20other%20factor-regulated%20calcium%20reabsorption&termId=550039897&source=kegg) | 3 | *Klk1b21, Klk1b9, Klk1b26* | 12.7 | 1.68 | 2.18 x 10^-2^ |
| [Aldosterone synthesis and secretion](https://david.ncifcrf.gov/kegg.jsp?path=mmu04925$Aldosterone%20synthesis%20and%20secretion&termId=550039890&source=kegg) | 3 | *Cyp11a1, Hsd3b1, Hsd3b6* | 7.70 | 1.68 | 4.20 x 10^-2^ |

**Table S8.** **The pathway analysis of the DEGs in round spermatids between P63^(+/-)^ mice and wild type mice**

| **Pathway** | **Sequence in Biological Process** | **Gene Name** | **Fold Enrichment** | **Percentage of genes in list** | **P Value** |
| --- | --- | --- | --- | --- | --- |
| [TNF signaling pathway](https://david.ncifcrf.gov/kegg.jsp?path=mmu04668$TNF%20signaling%20pathway&termId=550039855&source=kegg) | 14 | *Cebpb, Fos, Traf2, Creb3l2, Creb3l3, Ccl2, Csf1, Icam1, Junb, Jun, Lif, Map3k14, Tnfrsf1b, Vcam1* | 2.99 | 1.22 | 7.40 x 10^-4^ |
| [PI3K-Akt signaling pathway](https://david.ncifcrf.gov/kegg.jsp?path=mmu04151$PI3K-Akt%20signaling%20pathway&termId=550039818&source=kegg) | 28 | *Crtc2, Ddit4, Fasl, Brca1, Creb3l2, Creb3l3, Col4a1, Col4a3, Col4a5, Col5a1, Col5a2, Col27a1, Csf1, Fgf11, Fn1, Itga5, Itga7, Kit, Lamb1, Lama2, Lamb2, Nos3, Osm, Pgf, Pdgfra, Pdgfa, Ywhag, Vegfa* | 1.85 | 2.43 | 2.20 x 10^-3^ |
| [MAPK signaling pathway](https://david.ncifcrf.gov/kegg.jsp?path=mmu04010$MAPK%20signaling%20pathway&termId=550039790&source=kegg) | 18 | *Fos, Fasl, Mos, Traf2, Cacna2d2, Fgf11, Gadd45a, Gadd45b, Jun, Mapk8ip3, Map3k1, Map3k14, Map4k4, Nlk, Pdgfra, Pdgfa, Zak, Tgfb3* | 1.65 | 1.56 | 4.37 x 10^-2^ |
|  |  |  |  |  |  |
| [Hippo signaling pathway](https://david.ncifcrf.gov/kegg.jsp?path=mmu04390$Hippo%20signaling%20pathway&termId=550039832&source=kegg) | 14 | *Tead1, Tead1, Actg1, Apc, Bmpr2, Ctnna1, Itgb2, Llgl1, Tcf7l1, Tcf7, Trp53bp2, Trp73, Tgfb3, Ywhag, Wnt3a* | 2.16 | 1.22 | 1.27 x 10^-2^ |
| [Regulation of actin cytoskeleton](https://david.ncifcrf.gov/kegg.jsp?path=mmu04810$Regulation%20of%20actin%20cytoskeleton&termId=550039874&source=kegg) | 19 | *Iqgap3, Mos, Rock2, Wasl, Arpc5, Actg1, Apc, Bcar1, Fgf11, Fn1, Gsn, Itga5, Itga7, Itgb2, Mylk4, Mylk, Pdgfra, Pdgfa, Ssh3* | 2.16 | 1.65 | 4.69 x 10^-3^ |

**Supplemental Figures**

**Supplemental Figure 1**


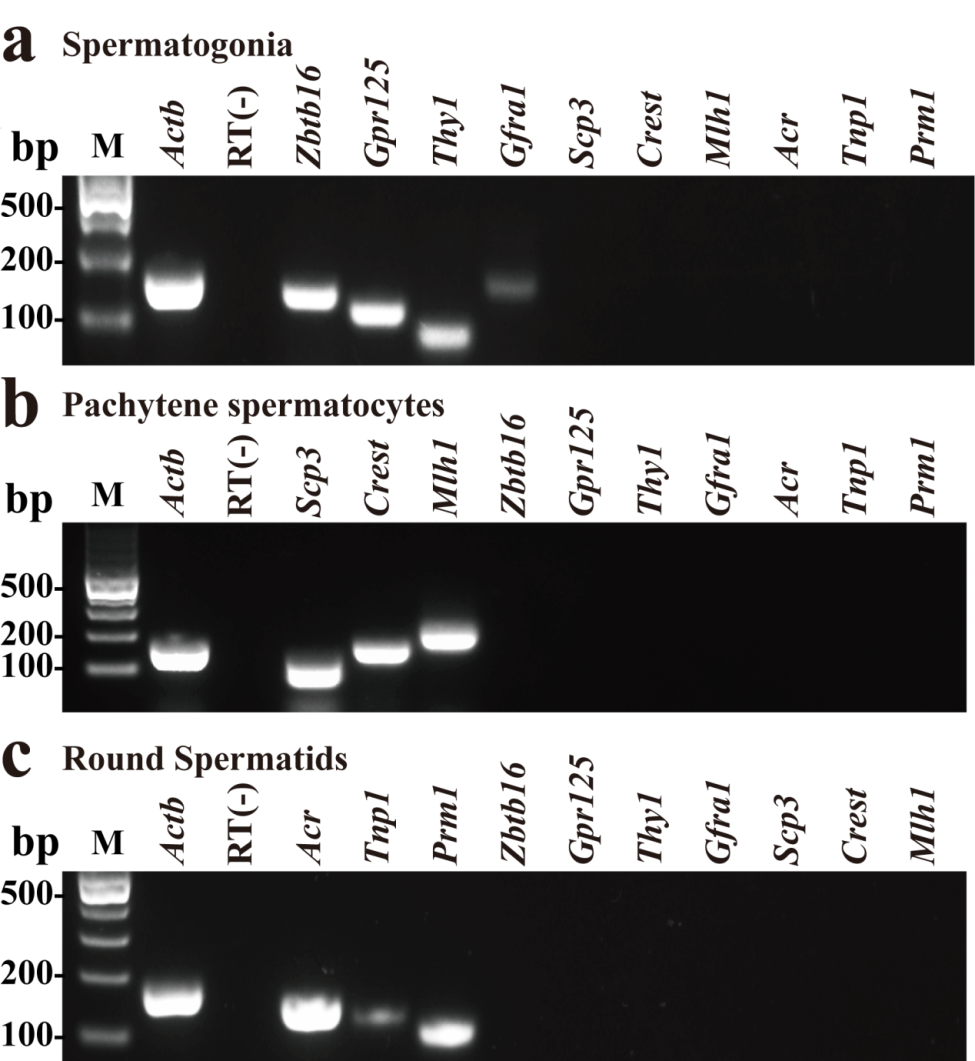


**Supplemental Figure 2**

**
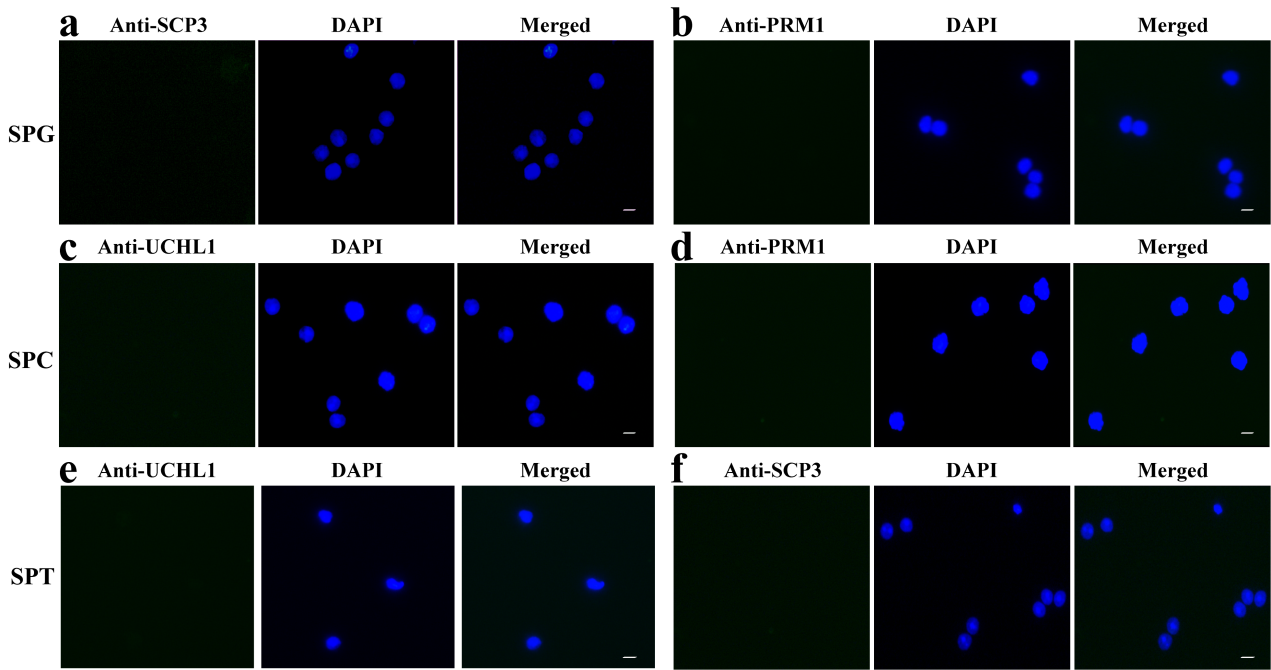
**

**Supplemental Figure 3**

**
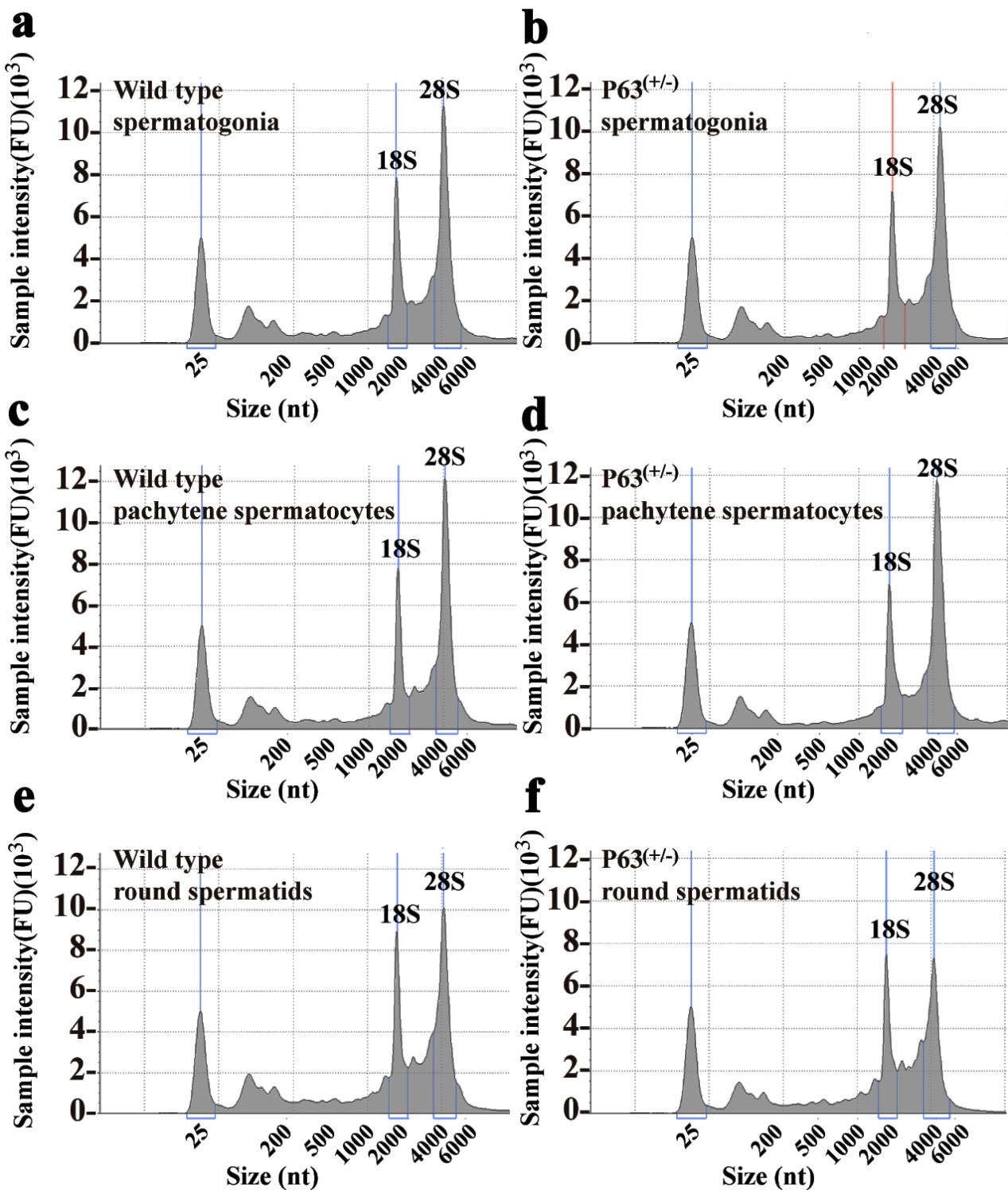
**

**Legends for Supplemental Figures**

**Supplemental Figure 1**. **Verification of the purities of the isolated spermatogonia, pachytene spermatocytes and round spermatids at the transcriptional level.** RT-PCR showed that the transcription of *Zbtb16*, *Gpr125*, *Thy1* and *Gfra1* was present in the isolated spermatogonia, whereas *SCP3*, *Crest*, *Mlh1*, *Acr*, *Tnp1* and *Prm1* were not detected (**a**). *SCP3*, *Crest* and *Mlh1* were detected in the isolated pachytene spermatocytes; in contrast, *Zbtb16*, *Gpr125*, *Thy1*, *Gfra1*, *Acr*, *Tnp1* and *Prm1* were not detected in these cells (**b**). *Acr*, *Tnp1* and *Prm1* were found in the isolated pachytene spermatocytes; conversely, *Zbtb16*, *Gpr125*, *Thy1*, *Gfra1*, *Acr*, *Tnp1* and *Prm1* were not detected in these cells (**c**). *Actb* was employed as a loading control of total RNA, and RNA samples without RT (RT-) but with PCR by *Actb* primers were used as negative controls.

**Supplemental Figure 2**. **Confirmation of the purities of the isolated spermatogonia, pachytene spermatocytes and round spermatids at the translation level.** Immunocytochemistry demonstrated absence of SCP3 (**a**) and PRM1 (**b**) proteins in the isolated spermatogonia (SPG). The proteins of UCHL1 (**c**) and PRM1 (**d**) were not expressed in the isolated pachytene spermatocytes (SPC). In addition, the expression of UCHL1 (**e**) and SCP3 (**f**) were undetected in the isolated round spermatids (SPT). Scale bars in **a-f**= 10 μm.

**Supplemental Figure 3**. **The quality assessment of total RNA from spermatogonia, pachytene spermatocytes and round spermatids in P63^(+/-)^ and wild type mice.** Electropherogram by Agilent bioanalyzer displayed the concentrations and nucleotides (nt) sizes of RNAs for spermatogonia of wild type mice **(a)** and P63^(+/-)^ mice **(b)**, pachytene spermatocytes of wild type mice **(c)** and P63^(+/-)^ mice **(d)**, and round spermatids of wild type mice **(e)** and P63^(+/-)^ mice **(f)**.
